# Regulatory roles of Osteopontin in lung epithelial inflammation and epithelial‐telocyte interaction

**DOI:** 10.1002/ctm2.1381

**Published:** 2023-08-21

**Authors:** Huirong Fu, Xuanqi Liu, Lin Shi, Lingyan Wang, Hao Fang, Xiangdong Wang, Dongli Song

**Affiliations:** ^1^ Department of Pulmonary and Critical Care Medicine Zhongshan Hospital Fudan University Shanghai Medical College Shanghai China; ^2^ Center for Tumor Diagnosis & Therapy Jinshan Hospital Fudan University Shanghai Medical College Shanghai China; ^3^ Shanghai Institute of Clinical Bioinformatics Shanghai China; ^4^ Shanghai Engineering Research for AI Technology for Cardiopulmonary Diseases Shanghai China; ^5^ Department of Anesthesiology Zhongshan Hospital Fudan University Shanghai Medical College Shanghai China; ^6^ Department of Pulmonary Medicine Shanghai Xuhui Central Hospital Fudan University Shanghai China; ^7^ Department of Anesthesiology Shanghai Geriatric Medical Center Shanghai China

**Keywords:** cell‐cell interaction, epithelial cells, inflammation, lung, Osteopontin, telocytes

## Abstract

**Background:**

Lung epithelial cells play important roles in lung inflammation and injury, although mechanisms remain unclear. Osteopontin (OPN) has essential roles in epithelial damage and repair and in lung cancer biological behaviours. Telocyte (TC) is a type of interstitial cell that interacts with epithelial cells to alleviate acute inflammation and lung injury. The present studies aim at exploring potential mechanisms by which OPN regulates the epithelial origin lung inflammation and the interaction of epithelial cells with TCs in acute and chronic lung injury.

**Methods:**

The lung disease specificity of OPN and epithelial inflammation were defined by bioinformatics. We evaluated the regulatory roles of OPN in OPN‐knockdown or over‐expressed bronchial epithelia (HBEs) challenged with cigarette smoke extracts (CSE) or in animals with genome OPN knockout (*gKO*) or lung conditional OPN knockout (*cKO*). Acute lung injury and chronic obstructive pulmonary disease (COPD) were induced by smoking or lipopolysaccharide (LPS). Effects of OPN on PI3K subunits and ERK were assessed using the inhibitors. Spatialization and distribution of OPN, OPN‐positive epithelial subtypes, and TCs were defined by spatial transcriptomics. The interaction between HBEs and TCs was assayed by the co‐culture system.

**Results:**

Levels of OPN expression increased in smokers, smokers with COPD, and smokers with COPD and lung cancer, as compared with healthy nonsmokers. LPS and/or CSE induced over‐production of cytokines from HBEs, dependent upon the dysfunction of OPN. The severity of lung inflammation and injury was significantly lower in OPN‐*gKO* or OPN‐*cKO* mice. HBEs transferred with OPN enhanced the expression of phosphoinositide 3‐kinase (PI3K)CA/p110α, PIK3CB/p110β, PIK3CD/p110δ, PIK3CG/p110γ, PIK3R1, PIK3R2 or PIK3R3. Spatial locations of OPN and OPN‐positive epithelial subtypes showed the tight contact of airway epithelia and TCs. Epithelial OPN regulated the epithelial communication with TCs, and the down‐regulation of OPN induced more alterations in transcriptomic profiles than the up‐regulation.

**Conclusion:**

Our data evidenced that OPN regulated lung epithelial inflammation, injury, and cell communication between epithelium and TCs in acute and chronic lung injury. The conditional control of lung epithelial OPN may be an alternative for preventing and treating epithelial‐origin lung inflammation and injury.

## Graphical abstract

1. Alterations of potential target gene expression in the database.

2. Genetic modification and/or external Osteopontin (OPN) proteins in cells and animals demonstrated the role of OPN and the mechanisms of Akt and extracellular‐signal‐regulated kinase signalling.

3. Spatial distribution of OPN‐enriched lung epithelia and telocytes indicated their interaction.

4. Regulatory roles of OPN in cell‐cell interactions were further demonstrated by the cell co‐culture system.

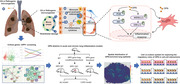

## 1 INTRODUCTION

Lung inflammation is a critical primary and/or secondary process of acute and chronic lung injuries and an initial factor for microenvironmental changes during the development of lung cancer and fibrosis.^1^ Of multiple lung resident cells, epithelial cells respond as the major receptor to external pathogens and secondarily release various cytokines to induce lung inflammation.^2^ Activated lung epithelial cells contribute to lung injury by altering mitochondrial function and mitophagy, production of cytokines/chemokines and radial oxygen species, and proteomic and lipidomic profiles.^3^, ^4^, ^5^ Smoking or lipopolysaccharide (LPS) could lead to the alteration of genes involved in lipid metabolism in airway epithelial cells and enhance the production of inflammatory mediators through the STAR‐related lipid transfer domain‐3 and mitofusin 2 pathway, cholesterol dysfunction, or disorder of mitochondrial function and dynamics.^6^

Osteopontin (OPN, also named SPP1), a highly phosphorylated glycophosphoprotein, has acidic characteristics enriched with aspartic acids and plays multiple functions which are highly dependent upon cell types and locations. Of those roles, OPN can stimulate the production of many inflammatory mediators, activate inflammatory cells, and promote the progress of inflammation in tissue injury, dysmetabolism, or carcinogenesis.^7^ OPN plays important regulatory roles in multiple pathological processes by inducing cell interactions and epithelial‐to‐mesenchymal transitions and altering cell sensitivity to pathogens and therapies.^8^, ^9^ By integrating multi‐omics with clinical phenomics, circulating levels of OPN were selected as a biomarker for acute exacerbation of chronic obstructive pulmonary disease (COPD).^1^ Plasma OPN was also proposed as a biomarker of coronavirus disease 2019 severity and multisystem inflammatory condition in children.^10^ These studies indicated that OPN plays an important role in acute and chronic lung injury, although molecular mechanisms remain unclear.

Telocytes (TCs) are a type of interstitial cell that can interact with epithelial cells to alleviate acute inflammation and lung injury. The present studies aim at investigating the regulatory roles of intra‐epithelial cell OPN in airway epithelial origin inflammation and epithelial‐telocyte interaction. We first evaluated dynamical changes of OPN between healthy and smokers, in smokers with or without COPD, COPD smokers with or without lung cancer, and patients with pneumonia, and then defined the specificity of OPN changes among lung tissues of patients with various lung diseases. The potential mechanisms were investigated in cigarette smoking extracts (CSE)‐induced OPN alterations in human bronchial epithelia (HBEs). Regulatory roles of intra‐epithelial OPN or exogenous OPN proteins in epithelial origin inflammation induced by CSE and/or lipopolysaccharide (LPS) were explored in OPN‐knockdown or over‐expressed HBEs or animals with genome OPN knockout (*gKO*) or lung conditional OPN knockout (*cKO*). We assessed the involvement of phosphoinositide 3‐kinase (PI3K) subunits and extracellular‐signal‐regulated kinase (ERK) in OPN‐dependent epithelial response to CSE or LPS as well as the potential mechanism by which external OPN‐induced epithelial inflammation. The spatialization of OPN expression and lung epithelial subtypes as well as TCs, including OPN‐positive alveolar type 1 and 2, mucous epithelia, basal, club, ciliated and Goblet cells, were evaluated by spatial transcriptomics. The epithelial‐telocyte interaction was demonstrated by the co‐culture system. The mechanisms by which the intra‐epithelial OPN regulates epithelial‐telocyte interactions were evaluated by transcriptomic analyses.

## 2 METHODS

### 2.1 COPD bioinformatics analysis

Available data from public databases contained lung single‐cell transcriptomic profiles of healthy nonsmokers (H.NS), healthy smokers (H.S), smokers with COPD (S.COPD) and smokers with both COPD and squamous cell carcinoma (SCC) (S.COPD.SCC) and were downloaded from Gene Expression Omnibus (GEO) of the National Center for Biotechnology Information website. Raw data normalization was processed with a robust multi‐array average (RMA) approach.^11^ Batch effects were removed using ComBat.^12^ Differentially expressed genes (DEGs) were identified by limma.^13^ Protein‐protein interaction (PPI) information for OPN and lactotransferrin (LTF) was retrieved by using Search Tool for the Retrieval of Interacting Genes/Proteins (STRING).^14^

### 2.2 Cell culture and reagents

HBEs, immortalized cell lines (CRL‐2741) from American Type Culture Collection (ATCC), and A549 cells, human lung adenocarcinoma cells, were cultured in RPMI 1640 (12633012; ThermoFisher) contained 10% fetal bovine serum (A3161001C; ThermoFisher). Isolated primary human lung TCs were immersed totally with TCs special protein antibodies and identified with vimentin, platelet‐derived growth factor (PDGFα) and forkhead box L1 (FOXL1) with DAPI for nucleus (detailed information in Table S1).^15^, ^16^ The images were produced with a confocal microscope.

HBEs and TCs were co‐cultured in the transwell system with a 0.4‐μm membrane. About 5 × 104 TCs and HBEs were planked at the bottom of the plate and at the upper chamber (140620; ThermoFisher), respectively. LY294002 (HY‐10108) and GDC‐0994 (HY‐15947) were obtained from MedChemExpress. Transfection reagents Lipofectamine 2000 (11668019) and Lipofectamine 3000 (L3000008) were purchased from ThermoFisher Scientific. All in vitro experiments were conducted and triplicated with six per group in each experiment.

### 2.3 Production of CSE

The CSE was prepared according to the method, as reported previously.^8^ A pruned straw was connected with a syringe using a rubber band to make a smoking device. The cigarette smoking device is used to draw smoke from one end of a cigarette removing the filter tip. The smoke was injected into a 50 mL centrifuge tube containing 5 mL RPMI 1640 medium fully shaken with 10% fetal bovine serum and 1% penicillin‐streptomycin. After the repeating, the initial CSE was obtained from the smoke of the exhausted cigarette, filtered, and sterilized. The absorbance value of filtered initial CSE fully dissolved in RPMI 1640 culture medium was measured with the microplate reader at the wavelength of 320 nm. The filtered initial CSE was diluted to the absorbance value of 1.158 as 100% CSE.

### 2.4 Cell proliferation

HBEs were treated with vehicle or 2%, 4%, 6%, 8% or 10% CSE for 24 h, respectively. The cell proliferation was assessed with Cell Counting Kit‐8 (CCK‐8, C0043; Beyotime). HBEs were incubated for different time points, washed before adding 200 μl of CCK‐8 reagent, and maintained at 37°C for 2 h. The absorbance value at 450 nm wavelength was analyzed with a microplate reader (Multiskan FC; ThermoFisher).

### 2.5 Quantitative real‐time polymerase chain reaction

Total RNA from cultured cells or mouse lung tissue lysis was separated with TRIzol reagent (15596026; Invitrogen) and was reverse‐transcribed with reverse transcription reagents (RR036A; Takara). Quantitative real‐time polymerase chain reaction (qRT‐PCR) proceeded with ABI 7000 PCR (Eppendorf) using an SYBR Green Master reagent (RR420Q; Takara). The relative gene mRNA expression was expressed by the comparative formula method (2 ^−ΔΔCt) using ACTB as a housekeeping gene. The information on gene primers is detailed in Table S2.

### 2.6 Western blot

Cell and lung tissue proteins were obtained with a protein extraction kit (KGP1100; Keygen Biotech,) and measured with a BCA test kit (PC0020; Solarbio). The 30ug protein was gently loaded to 10% SDS‐PAGE, transferred to the PVDF membrane, and blocked with 5% BSA (SW3015; Solarbio) for an hour. The membrane was soaked with corresponding primary antibodies against OPN, p‐Akt, Akt, p‐ERK, ERK, tumour necrosis factor‐alpha (TNFα), and GAPDH (Table S1) at 4°C overnight. Horseradish peroxidase‐conjugated corresponding secondary antibodies were immersed for an hour. Bands were shown using ECL and quantified using ImageJ software.

### 2.7 Preparation of siRNA

The highly effective sequences were selected after testing of small interference RNA (siRNA) from GenePharma, as shown below: OPN sense‐CCACAGUAGACACAUAUGATT, antisense‐UCAUAUGUGUCUACUGUGGTT; negative control: UUCUCCGAACGUGUCACGUTT, antisense‐ACGUGACACGUUCGG AGAATT.

### 2.8 Preparation of short hairpin RNA

A PHY‐008 vector with the OPN cDNA was constructed by Genechem to form the OPN expression system. The short hairpin (shRNA) contained a GV112 vector with the targeting sequence CCACAAGCAGTCCAGATTATA (shRNA‐3) or ACGAGTCAGCTGGATGACC (shRNA‐4) for OPN and was established by Genechem.

### 2.9 CRISPR/Cas9 system

The gRNA sequence was designed using a gRNA design tool website (https://crispr.mit.edu/). The cassette of pCas‐GuideEF1a GFP vector with a designed sequence (ATCAGAGTCGTTCGAGTCAA) was formed to develop the transcription progress of the gRNA. This designed sequence fulfilled with high activity of on‐target to promote expression progress of the Cas9 protein.

### 2.10 Spatial transcriptome analyses

Cancer and para‐cancerous samples (*n* = 4 pairs) from three lung cancer patients (Zhongshan Hospital of Fudan University). On the basis of the 2015 WHO classification, non‐small cell lung cancer (NSCLC) was diagnosed and graded. This research was consented to by the Ethics Committee of Zhongshan Hospital of Fudan University (B2021‐265) and the patients. The diagnosis was finally verified with a histological examination. Cancer and para‐cancerous samples (> 2 cm from cancer edge) were harvested, collected, transported, and restored, according to clinical sampling procedures for spatial transcriptomics reported previously and detailed experimental steps referred to in our previous studies.^17^

The data of lung cancer samples contained 1246 genes measured on 1888 per spot, 4378 genes on 3256 spots, and 5642 genes on 2488 spots, respectively. The data of the para‐cancer sample contained 2090 genes measured on 1862 spots. In the analysis, we validated the transcriptomic expression and spatial location of OPN in lung tissues. Of tissue cells, the spatial distribution of OPN‐positive lung epithelial subtypes (e.g. AT1, AT2, mucous, basal, club, ciliated and goblet epithelia) was analyzed and visualized, respectively, by mapping the appearance of both positive OPN and each lung epithelial subtype gene marker panel, for example, AGER, HOPX, PDPN, CLDN18 and CLIC5 for alveolar epithelial type 1; MUC1, SFTPB, SFTPC, ETV5 and SFTPD for alveolar epithelial type 2; KRT5 and KRT14 for basal epithelia; FOXJ1 marker for ciliated epithelia; SCGB1A1 and SCGB3A2 for club epithelia; MUC5B with MUC5AC for goblet epithelia; and MUC5AC and MUC5A for mucous epithelia. The log2 feature max value of gene expression was presented in the spot plot.

### 2.11 Cell bulk RNA sequencing

RNA amount and integrity were measured with RNA Nano 6000 Assay Kit of the Bioanalyzer 2100 System (Agilent Technologies) and cleaned with poly‐T oligonucleotide‐attached magnetic beads. 370–420 bp screened cDNA was applied for PCR amplification, the product with AMPure XP beads (Beckman Coulter) was cleaned, and the library was collected. qRT‐PCR was used to quantify the library using the Illumina NovaSeq 6000 platform to pool, target and sequence.

### 2.12 Validation of OPN gene expression in lung epithelial subtypes using scRNA‐seq

The expression of OPN in alveolar epithelial types I and II, mucous, basal, ciliated, Goblet, club, neuroendocrine or signalling AT2 epithelia was analyzed with datasets (GSE128169, GSE128033, GSE136831, GSE131907, E‐MTAB‐6653 and E‐MTAB‐6149). By combining integration anchors and hypervariable genes, data were normalized and integrated to proceed with the standardization, dimensionality down‐regulation and clustering. The data set was annotated by the Seurat mapping references. The expressions of OPN in different epithelia between healthy (normal lung tissues) 20 samples or patients with COPD 15 samples were analyzed.

### 2.13 Validation of OPN gene expression in lung tissues using bulk RNA‐seq

The expression of genes in lung tissues at various pathophysiological stages was analyzed between healthy non‐smokers, smokers with or without COPD and COPD smokers with or without lung cancer, using bulk RNA‐seq (GSE5058, GSE8545, GSE11784, GSE11906 and GSE20257). The platforms of these five datasets are GPL570 [HG‐U133_Plus_2] Affymetrix Human Genome U133 Plus 2.0 Array. Sources are airway epithelial cells obtained by bronchoscopy and brushing in GSE5058, GSE8545, GSE11906 and GSE20257 datasets, while small airway epithelium in GSE11784. The study included 181 non‐smokers, 205 smokers and 95 COPD smokers (https://www.ncbi.nlm.nih.gov/geo/).

### 2.14 Reactive oxygen species production

The reactive oxygen species (ROS) production was measured by fluorescent DCHF‐DA assay reagent (S0033M; Beyotime). Resuspended HBEs were collected from a 6‐well plate after digestion, and incubated with fluorescent probe DCFH‐DA reagent for 20 min. Flow Cytometer was used to measure the intracellular fluorescence intensity.

### 2.15 Measurement of mitochondrial membrane potential

Collected HBEs were incubated with MitoTracker Red CMXRos staining solution for 0.2 μM at 37°C for 30 min.^18^ The intracellular fluorescence intensity was counted by Flow Cytometer. The mitochondrial membrane potential (MMP) was calculated using mean fluorescence intensity.

### 2.16 Measurement of apoptosis

Trypsin‐digested cells were collected and resuspended with Annexin V‐FITC and propidium iodide (PI), respectively (C1062M; Beyotime). Cell apoptosis was defined and analyzed using flow cytometry.

### 2.17 Assess glutathione

The plasma levels of glutathione (GSH) were assessed after centrifugation at 3500 rpm/min for 10 min under the assay protocol (A006‐2; Jiancheng). The values were obtained with a microplate reader under 405 nm.

### 2.18 Lung tissue pathology

The lung tissue samples were fixed at 4% paraformaldehyde, embedded with paraffin, and sectioned. Tissue sections at 5 μm were covered with Hematoxylin and Eosin (H&E) and the images were collected. The mean linear intercept length was used to estimate the alveolar enlargement with digital imaging software (Image J).^19^ The severity of lung injury was scored on the basis of the ATS report.^20^

### 2.19 Immunohistochemistry

Cells were fixed using 4% paraformaldehyde and lung tissues were fixed and embedded with paraffin, and incubated with antibodies against OPN (ab214050; Abcam) and Prosurfactant Protein C (ab90716; Abcam), followed by staining with fluorescein‐linked anti‐rabbit antibody (A0516; Beyotime). The cell nuclei were stained with DAPI.

The expression of OPN proteins was detected and measured by immunohistology. Sections were incubated with the primary antibody (ab214050; Abcam) at 4°C overnight and covered with the corresponding secondary antibody (ab205718; Abcam). Sections were stained with diaminobenzidine (DAB) and counterstained with hematoxylin. The ratio of integral optical density (IOD) of OPN in the area was Mean Density.^21^ Images were analyzed with Aipathwell.

### 2.20 TUNEL assay

The TUNEL‐positive cells were tested using Tunel Cell Apoptosis Detection Kit (G1501‐50T; Servicebio) and observed with fluorescence microscopy.

TUNEL‐positive cells were counted in each field.

### 2.21 Cytokine protein measurement

The concentrations of interleukin (IL)‐6, IL‐8, IL‐1β, and TNFα proteins in cell culture supernatants and mouse cytokines such as IL‐6 and CXCL1^22^, ^23^ from bronchoalveolar lavage fluid (BALF) supernatants and circulating plasma were measured by enzyme‐linked immunosorbent assay (ELISA) kits under the manufacturer's protocol. These ELISA kits were human IL‐6 (D6050; R&D Systems), human IL‐8 (D8000C; R&D Systems), human IL‐1β (DLB50; R&D Systems), human TNFα (DTA00D; R&D Systems), mouse IL‐6 (M6000B; R&D Systems), and mouse CXCL1 (MKC00B; R&D Systems).

### 2.22 Lung conditional *SPP1* knockout animals

Animals were kept under specific pathogen‐free conditions with free access to sterile food and water. The conditional lung epithelial cells specific SPP1 knockout (*cKO*) mice were established with C57BL/6J background. Mice of *Sftpc^CreERT2^* (Jackson Laboratory, Maine, US) and SPP1^flox/flox^ genotype were crossed to generate *Sftpc^CreERT2^*; SPP1^flox/flox^ (SPP1‐cKO) mice were generated and validated (Cyagen Biosciences Inc). Eight to 16‐week‐old mice received intraperitoneal injections with tamoxifen (1 mg/mL; T5648, Sigm‐Aldrich) to induce Cre recombinase activity. The animal experiment procedures in the study were authorized by the Animal Ethics Committee of Zhongshan Hospital of Fudan University (SCXK(Hu)2012−0002). Littermates represented wild‐type (WT) mice were used as controls.

### 2.23 Genome *SPP1* knockout animals

C57BL/6J ‐*SPP1* deficient mice (*SPP1‐gKO*, B6. Cg‐Spp1tm1blh/J) 8‐ to 12‐week‐old were purchased from the Jackson Laboratory and maintained in the animal facility with free access to drinking water and chow. All mice were kept with a light‐dark cycle of 12 h, under specific pathogen‐free conditions.

### 2.24 COPD model

C57BL/6, wild‐type mice, SPP1‐cKO mice, and SPP1‐gKO mice were exposed to room air or cigarette smoke (CS) by using a Teague TE‐10z device for 1 h daily and 5 days a week for 12 weeks, containing the total particulate matter delivered changing from 150 and 200 mg/m to induce a COPD model.^6^, ^8^ Pathological characters of COPD model included enlarged alveolar spaces, lung parenchymal emphysema, destroyed alveolar walls, and airway wall thickening of the lung via HE staining.

### 2.25 Acute lung injury model

Mice were injected intratracheally with LPS at 3 mg/kg body weight for 24 h to induce acute lung injury (ALI), after which mice were sacrificed, and the blood, BALF and lung tissues were collected for further analysis. Pathological changes of lung tissues revealed that alveolar wall rupture, tissue oedema and red blood cells increased in the alveoli and leukocyte infiltration in the alveolar wall in animals with ALI.^24^

### 2.26 Statistics

All data are shown as the mean ± SEM. The data were calculated via SPSS 21.0 (SPSS) software. Statistical comparisons were analyzed by one‐way analysis of variance (ANOVA) with Tukey's post hoc test and two‐way ANOVA with Tukey's post hoc test. The significance was defined as *p* < .05.

## 3 RESULTS

### 3.1 Validation of OPN expression in COPD and smoking

**FIGURE 1:**
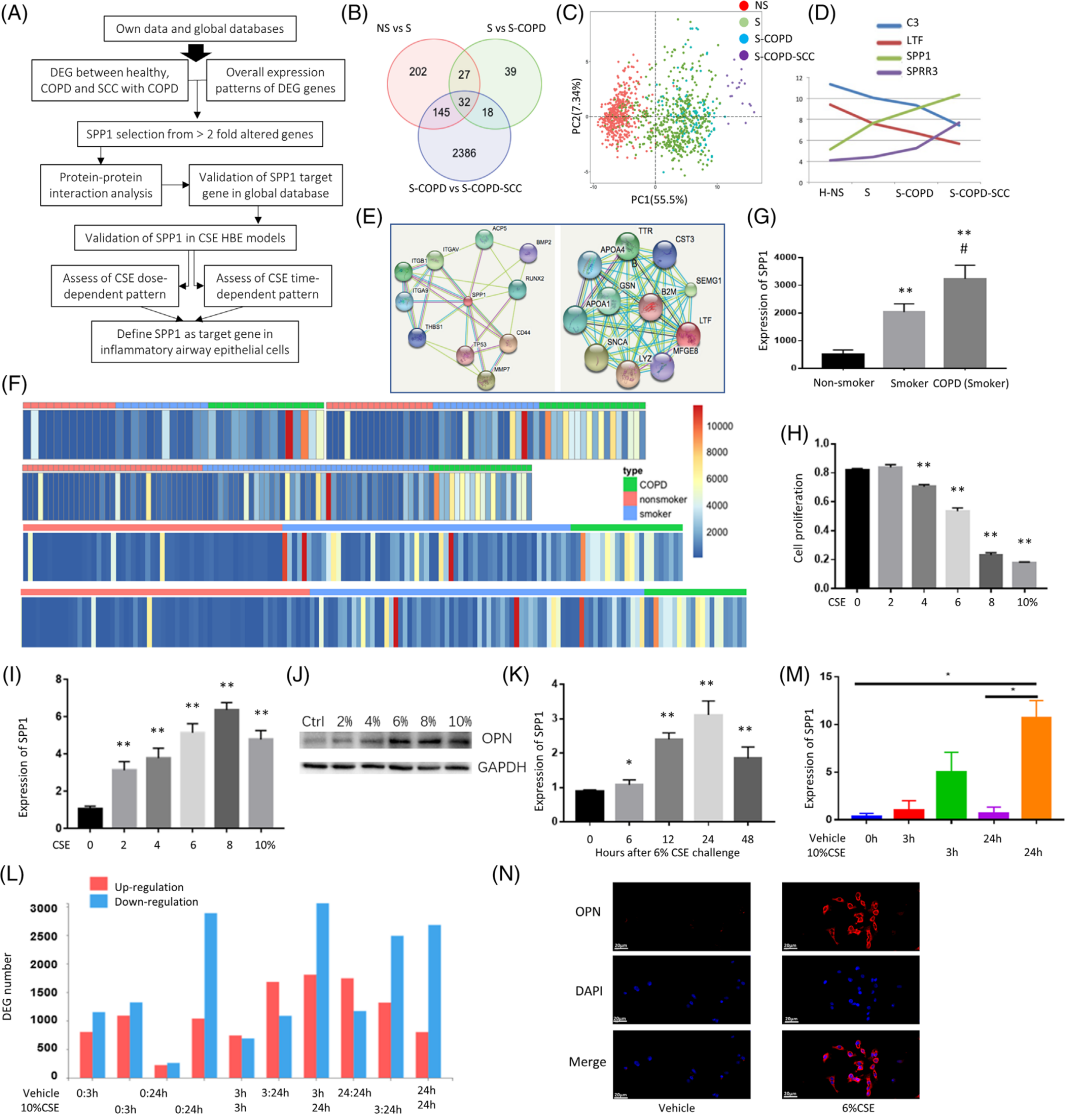
Target validation by clinical preclinical studies. Bioinformatics analysis for *SPP1* gene in COPD patients from a public database and dynamic expression of SPP1 in human normal airway epithelia (HBEs) after challenge with CSE at different concentrations. (A) Workflow of *SPP1* identification in lung epithelial cells. (B) The expression of *SPP1* mRNA at various pathophysiological stages of healthy nonsmokers (H.NS), healthy smokers (H.S), smokers with COPD (S.COPD) and COPD smokers with SCC (S.COPD.SCC). (C) Distribution of differentially expressed genes (DEGs) among stages of diseases. (D) The validations of *SPP1*, *LTS*, *C3* and *SPRR3* in lung epithelial cells. (E) *SPP1*‐ or *LTF*‐dominated transcriptomic networks. (F) *SPP1* mRNA expression of lung tissues from five public databases. (G) The expression of *SPP1* mRNA in smokers or smokers with COPD profile data from five databases. (H) Cell proliferation of HBEs after challenge with CSE at different concentrations. (I) *SPP1* expression in HBEs after challenge with CSE at different concentrations. (J) Levels of Osteopontin (OPN) proteins in HBEs after challenge with CSE at different concentrations. (K) *SPP1* expression in HBEs after challenge with 6% CSE in 48 h. (L) Changes of transcriptomic profiles in HBEs treated with vehicle or CSE. (M) *SPP1* mRNA alteration in HBEs after being treated with vehicle or CSE for 24 h. (N) OPN protein expression (red) in HBEs after being treated with 6% CSE. Nuclei were stained with DAPI (blue). * and ** stand for *p* < .05 and .01, as compared with negative controls and ^#^and ^##^for *p* < .05 and .01, as compared with positive controls.

We first mapped alterations of potential target gene expression in a proposed dynamic development of chronic lung inflammation from health to COPD and from COPD to lung cancer, defined the target gene like OPN (SPP1) dynamically and continuously increased, and validated the significance of OPN in lung epithelial cells, as detailed in Figure 1A. Since there is a direct correlation between smoking, COPD and lung cancer,^25^ we further evaluated the transcriptomic DEGs in lung tissue at various pathophysiological stages and found 202 DEGs between healthy non‐smokers and smokers, 39 between smokers with or without COPD, and 2386 between COPD smokers with or without (Figure 1B). Of those DEGs, 32 genes appeared in three comparisons, detailed in Table S3. A clear distribution of DEGs among stages was shown in Figure 1C. The top three genes gradually increased or decreased in the transit process between stages: a significant increase of OPN expression, while a decline of Complement 3 (C3) and Lactotransferrin (LTF) (Figure 1D). C3 interacts with other complement system proteins, mainly involved with the innate immune system.^26^ LTF possesses iron absorption and regulates antiviral and anti‐carcinogenic activities.^27^ Small proline‐rich protein 3 (SPRR3) constitutes cornified cell envelope precursors and participates in mucosal inflammation and barrier function.^28^ OPN increased significantly during the transit between health with or without smoking, smokers with or without COPD, and COPD smokers with or without SCC. OPN was chosen as the target to deeply explore the regulatory mechanisms of epithelial‐origin inflammation. After the screening, we analyzed OPN‐dominated transcriptomic networks, mainly including ITGs, ACP5, BMP2, RUNX2, THBS1, CD44 and TP53 in COPD smokers, different from those in smokers (Figure 1E). We validated the OPN expression of lung tissues from five public databases, including 181 health non‐smokers, 205 smokers, and 95 COPD smokers, as shown in Figure 1F. The OPN expression significantly increased in smokers or COPD smokers, as compared with that in non‐smokers (Figure 1G, *p* < .05 or less, respectively). OPN expression from each database was presented in Figure S1. OPN expression in COPD smokers was significantly higher than that in smokers. About 1552 or 488 AT1, 5249 or 248 AT2, 131 or 45 basal cells, 4840 or 986 ciliated cells, 334 or 131 Club cells, 380 or 19 goblet cells, 475 or 86 mucous cells, as well as 386 or 126 signaling_AT2 were identified in healthy or COPD, respectively. Of those epithelial subsets, we noticed that OPN expression was significantly higher in airway goblet cells of patients with COPD, as compared with the healthy (Figure S2).

To explore potential mechanisms in epithelial origin inflammation and function, we validated the dynamic expression of SPP1 in HBEs after challenge with CSE at different concentrations and found a dose‐dependent decrease of cell proliferation (Figure 1H) and an increase of SPP1 expression (Figure 1I) and OPN protein contents (Figure 1J). The expression of SPP1 significantly increased from 6 h after the CSE challenge and reached the peak at 24 h (Figure 1K). To validate the biology‐specific cell model, we compared transcriptomic profiles of HBEs 0, 3 and 24 h after challenges with vehicle or CSE and defined the difference between time points and between vehicle‐ and CSE‐challenged cells. The degree of transcriptomic profiles in HBEs with vehicle altered relatively smaller between time points, while obviously varied between time points of CSE and between vehicle‐ and CSE‐treated cells at 24 h (Figure 1L), as referred to in the published literature.^29^ SPP1 increased over time after the CSE challenge (Figure 1M, p < 0.05). Figure 1N presented OPN protein expression elevated after 6% CSE. The lung airspace enlarged significantly in mice with smoking by measuring alveolar mean linear intercept and the lung emphysematous changes were observed in Figure S3A. SPP1 and OPN expression increased in lung tissues harvested from CS‐induced mice (Figure S3B–D), as compared with mice with the vehicle. Inflammatory cells infiltrated, lung tissues damaged, and alveolar walls became thickened more obviously in LPS‐treated mice (Figure S4A). SPP1 and OPN expression increased significantly in the lung tissues of mice with LPS (Figure S4B–D).

### 3.2 Regulatory roles of OPN in epithelial inflammation

**FIGURE 2:**
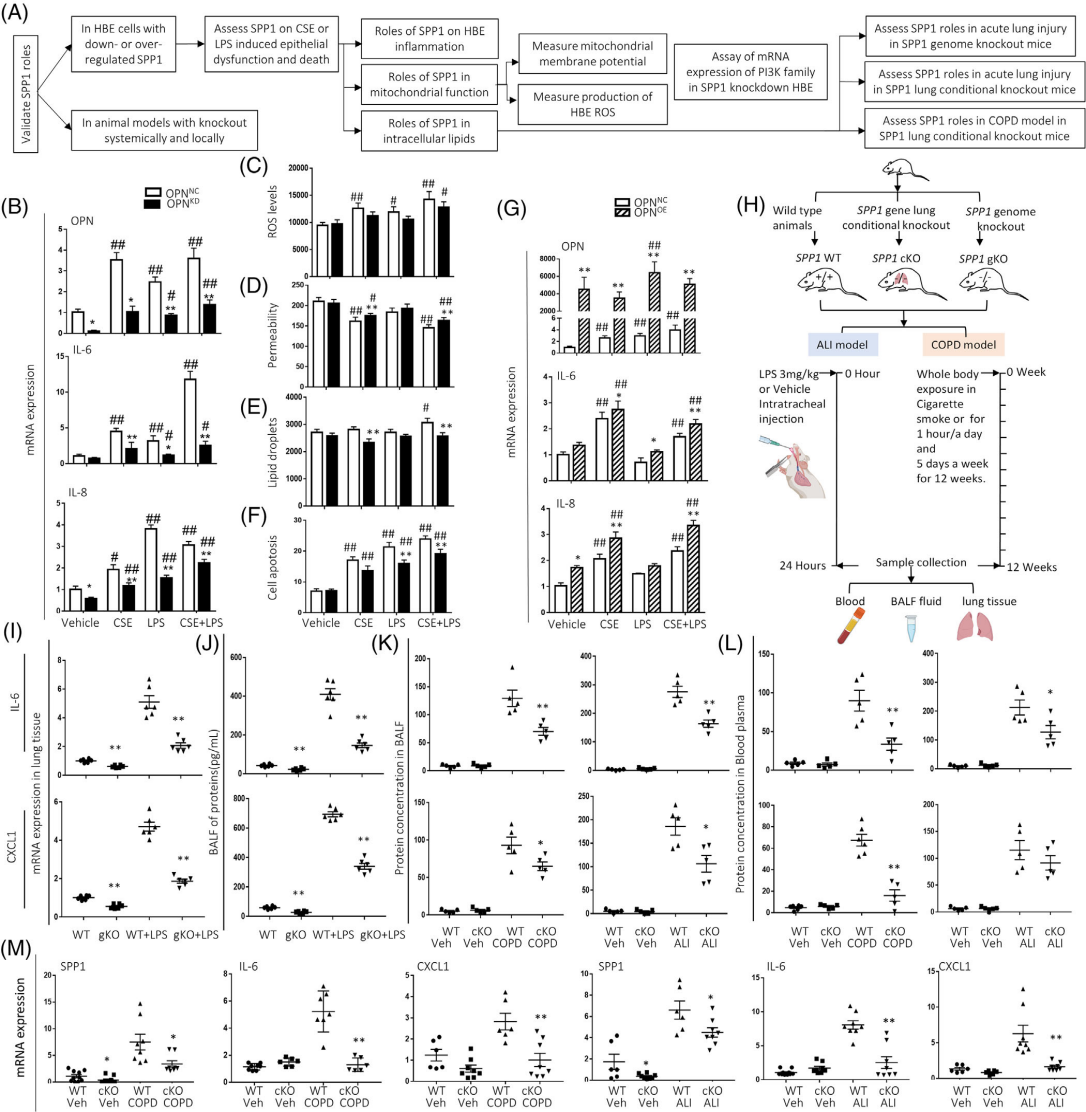
Effects of *SPP1* in human bronchial epithelia (HBEs) inflammation. (A) Schema diagram of regulatory roles of *SPP1* in HBEs inflammation. (B) *SPP1*, *IL‐6* and *IL‐8* mRNA expression of HBEs with negative control (*SPP1^NC^*) or knockdown *SPP1* gene expression (*SPP1^KD^*) after treatment with CSE, LPS, or CSE+LPS for 24 h. Reactive oxygen species (ROS, C), permeability of mitochondrial membrane (D), contents of lipid drops (E) and apoptotic rates (F) of *SPP1^NC^* or *SPP1^KD^* HBCs after treatment with CSE, LPS, or CSE+LPS for 24 h. (G) *SPP1*, *IL‐6* and *IL‐8* mRNA expression of *SPP1^NC^* or HBEs with knockdown *SPP1* gene expression (*SPP1^KD^*) after treatment with CSE, LPS or CSE combined with LPS for 24 h. (H) Workflow of acute lung injury (ALI) or chronic obstructive pulmonary disease (COPD) animal models of *Sftpc^CreERT2^* mice, conditional lung SPP1^flox/flox^ (SPP1‐cKO) mice, C57BL/6J ‐*SPP1* deficient mice (*SPP1‐gKO*), or widetype (WT) mice. (I) Levels of IL‐6 and CXCL1 mRNA expression in lung tissue of wildtype mice (WT) or C57BL/6J ‐*SPP1* deficient mice (*gKO*) mice with or without LPS. (J) Levels of IL‐6 and CXCL1 proteins in BALF from WT or *gKO* mice treated with or without LPS. Protein levels of IL‐6 and CXCL1 in BALF (K) and blood plasma (L) in WT or SPP1‐cKO mice with the vehicle, CSE‐induced COPD models, or LPS‐induced ALI models. (M) Levels of *SPP1*, *IL‐6* and *CXCL1* mRNA expression in WT or SPP1‐cKO mice with ALI or COPD (n = 5–8/group/time point). * and ** stand for *p* < .05 and .01, as compared with negative controls and ^#^and ^##^for *p* < .05 and .01, as compared with positive controls.

Our data demonstrated that CSE and LPS induced inflammation in the in vitro and in vivo models, similar to the previous findings.^30^, ^31^ We further evaluated roles and potential mechanisms of OPN in epithelial origin inflammation and function in HBEs or A549 cells with knockdown OPN gene expression (OPN*^KD^*) by OPN‐specific siRNA or shRNA, and in OPN‐knockout animals with organ‐non‐specific genome knockout or conditionally lung‐specific knockout (Figure 2A). OPN‐specific siRNAs or shRNAs had about 80−90% down‐regulated effects and were selected after the screening (Figure S5A), as compared with cells receiving negative control (OPN*^NC^*), of which shRNA‐3 was selected for the following study. Proinflammatory mediators (e.g. IL‐6, IL‐8, CXCL1 and MCP‐1) were secreted by bronchial epithelial cells after CSE or LPS stimulation.^32^, ^33^, ^34^ In HBEs, we noticed that IL‐6 and IL‐1β mRNA expression increased significantly in OPN*^NC^* cells with CSE, as compared with those in OPN*^NC^* with vehicle (Figure S3A). The treatment with CSE, LPS, or combination (CSE+LPS) induced a significant elevation of OPN mRNA expression in OPN*^NC^* cells, as compared with the corresponding OPN*^KD^* cells, of which CSE increased OPN expression more than LPS did (Figure 2B). OPN expression in OPN*^NC^* cells increased 3‐fold more than OPN*^KD^* cells after challenges with CSE or CSE+LPS (Figure 2B). CSE+LPS significantly increases IL‐6 mRNA expression in OPN*^NC^* cells at least 2‐fold higher than OPN*^NC^* and OPN*^KD^* cells with the vehicle, LPS or CSE alone, as well as OPN*^KD^* cells with CSE+LPS (Figure 2B). IL‐6 expression increased in OPN*^NC^* cells 5‐fold more than in OPN*^KD^* cells after CSE+LPS (Figure 2B). The expression of IL‐8 mRNA in OPN*^KD^* cells was significantly lower than those in OPN*^NC^* cells after challenges (Figure 2B, *p* < .05 or less, respectively), while IL‐8 levels in OPN*^KD^* cells with CSE+LPS were higher than those in OPN*^KD^* cells with CSE or LPS. IL‐8 expression in OPN*^NC^* cells increased about 3 folds more than in OPN*^KD^* cells after LPS treatment (Figure 2B). In A549 cells, we found that levels of IL‐6 and IL‐8 mRNA in OPN*^KD^* cells were almost 2 folds lower than in OPN*^NC^* with the vehicle. IL‐6 mRNA levels of OPN*^KD^* cells with CSE, LPS or CSE+LPS were significantly lower than those with vehicle (Figure S5C), and IL‐8 mRNA expression in OPN*^KD^* cells with LPS or CSE+LPS (Figure S5D). Levels of IL‐6, IL‐8 and MCP‐1 mRNA expression were also measured in A549 with OPN‐knockout (A549*^KO^*; Figure S5E).

Levels of intracellular ROS increased in OPN*^NC^* cells after challenges (Figure 2C and Figure S6A). The permeability of the mitochondrial membrane significantly declined in OPN*^NC^* cells with CSE or CSE+LPS, rather than in OPN*^KD^* cells (Figure 2D and Figure S6D). Contents of lipid drops significantly decreased in OPN*^KD^* cells with CSE or CSE+LPS, as compared with OPN*^NC^* cells (Figure 2E). Apoptotic rates significantly increased in OPN*^NC^* or OPN*^KD^* cells treated with CSE, LPS, or CSE+LPS, as compared with cells with vehicle (Figure 2F and Figure S6C), of which apoptotic rates of OPN*^KD^* cells with LPS or CSE+LPS were significantly lower than in OPN*^KD^* cells (*p* < .05 or less, respectively). In cells with OPN mRNA over‐expression (OPN*^OE^*), LPS induced higher expression of OPN in OPN*^OE^* cells than CSE or CSE+LPS did (Figure 2G). Expression of IL‐6 and IL‐8 mRNA significantly increased in OPN*^OE^* cells with challenges, as compared with that in OPN*^NC^* cells (Figure 2G).

### 3.3 Roles of OPN deletion in acute and chronic lung inflammation

We further evaluated the roles of OPN in the production of inflammatory mediators in mice with acute lung inflammation induced by intratracheally installing LPS for 24 h or chronic lung inflammation induced by CS for 12 weeks (Figure 2H). We induced acute lung inflammation in OPN‐*gKO* mice and observed the roles of OPN deletion in lung inflammation. We noticed that levels of IL‐6, CXCL1 and MCP‐1 mRNA expression (Figure 2I and Figure S5F) or proteins (Figure 2J and Figure S5G) in wildtype (WT) mice significantly elevated 24 h after LPS installation, as compared with OPN‐*gKO* mice, respectively. Plasma or BALF levels of those mediators in OPN‐*gKO* mice challenged with vehicle or LPS were significantly lower than in WT mice, respectively. To illustrate the local roles of lung OPN, we developed lung conditional OPN knockout (OPN‐*cKO*) mice and challenged mice with LPS for acute lung inflammation and with cigarette smoke for chronic lung inflammation (Figure 2H and Figure S7). We found that CS‐treated WT mice showed significant lung airspace increase, but not in OPN‐*cKO* mice (Figure S8A). Levels of GSH elevated significantly in OPN‐*cKO* mice, as compared with WT mice after CS treatment (Figure S8B). OPN‐*cKO* mice had less infiltration of inflammatory cells lung tissues and lung tissues compromised, as compared with LPS‐treated WT mice (Figure S9A). After LPS treatment, Levels of GSH were higher in OPN‐*cKO* mice than in WT mice (Figure S9B). Lung‐specific OPN deletion did not influence levels of IL‐6 and CXCL1 in BALF (Figure 2K) and plasma (Figure 2L) or mRNA in lung tissues (Figure 2M) of OPN‐*cKO* mice challenged with the vehicle, different from those in OPN‐*gKO* mice. Protein levels of IL‐6 in BALF and plasma of OPN‐*cKO* mice with COPD and ALI were significantly lower than those in WT mice with COPD or ALI, respectively. CXCL1 levels in BALF of OPN‐*cKO* mice with ALI (Figure 2K) and in plasma of OPN‐*cKO* mice with COPD (Figure 2L) were lower than WT mice with ALI and COPD. Levels of SPP1, IL‐6 and CXCL1 mRNA in lung tissues of OPN‐*cKO* mice with COPD and ALI were significantly lower than those of WT mice with COPD and ALI (Figure 2M). CS or LPS significantly induced more TUNEL‐positive cells in WT mice, but less in OPN‐*cKO* mice (Figures S8C and S9C).

### 3.4 The influence of OPN in PIK3 subunit expression

**FIGURE 3:**
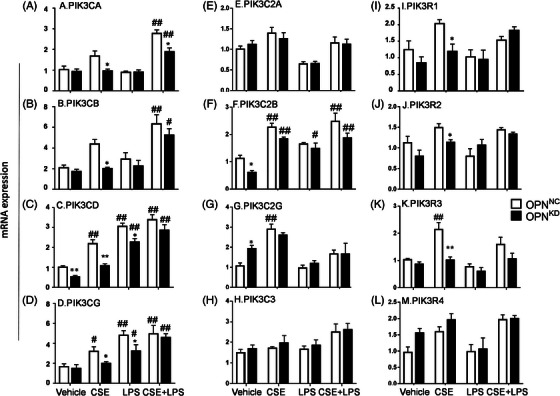
Roles of Osteopontin (OPN) in PI3K signals. The influence of *SPP1* in PIK3 subunit expression in human bronchial epithelia (HBEs) as negative controls *(SPP1^NC^*) or HBEs with *SPP1 siRNA (SPP1^KD^*) with LPS, CSE or CSE+LPS. mRNA expression levels of PIK3CA (A), PIK3CB (B), PIK3CD (C), PIK3CG (D), PIK3C2A (E), PIK3C2B (F), PIK3C2G (G), PIK3C3 (H), PIK3R1(I), PIK3R2 (J) and PIK3R3(K), and PIK3R4 (L) of *SPP1^NC^* and *SPP1^KD^* after treatment with CSE, LPS or combination for 24 h. * and ** stand for *p* < .05 and .01, as compared with negative controls and ^#^and ^##^for *p* < .05 and .01, as compared with positive controls.

PI3Ks contribute to multiple physiological and pathological processes within the airway, although the potential roles of PI3K subunits in lung inflammation and infection remain unclear. To define the involvement of PIK3 in OPN‐dependent responses, mRNA expression of 12 PIK3 subunit genes (i.e. PIK3CA, PIK3CB, PIK3CD, PIK3CG, PIK3C2A PIK3C2B, PIK3C2G, PIK3C3, PIK3R1, PIK3R2, PIK3R3 and PIK3R4) were measured. Of those, the expressions of PIK3CA (Figure 3A), PIK3CB (Figure 3B), PIK3CD (Figure 3C), and PIK3CG (Figure 3D) genes coded for the first‐class catalytic subunits p110α, p110β, p110δ and p110‐γ were altered significantly. The expression of PIK3CA and PIK3CB was higher in OPN*^NC^* cells with CSE and CSE+LPS, while PIK3CD and PIK3CG in CSE, LPS and CSE+LPS. Levels of PIK3CD and PIK3CG gene expression in OPN*^KD^* cells were significantly lower than those in OPN*^NC^* cells after those challenges, of which levels in LPS‐treated cells were higher than in CSE‐treated cells and levels in CSE+LPS‐treated cells were higher than either alone, respectively (Figure 3C,D). Of subunits of PIK3 catalytic subunit type 2 [i.e. PIK3C2A (Figure 3E), PIK3C2B (Figure 3F), PIK3C2G (Figure 3G), and PIK3C3 (Figure 3H)], the expression of PIK3C2B in cells with challenges was higher than in cells with the vehicle. Of PIK3 regulatory subunits, the expression of PIK3R1, PIK3R2, and PIK3R3 mRNA was significantly lower in OPN*^KD^* cells with CSE, as compared with the corresponding OPN*^NC^* cells (Figure 3I–K). The down‐regulation of OPN genes per se resulted in the reduction of PIK3CD (Figure 3C), and PIK3C2B (Figure 3F) expression, while the elevation of PIK3C3 (Figure 3H) and PIK3R4 (Figure 3L) expression.

### 3.5 Roles of external OPN in epithelial inflammation

**FIGURE 4:**
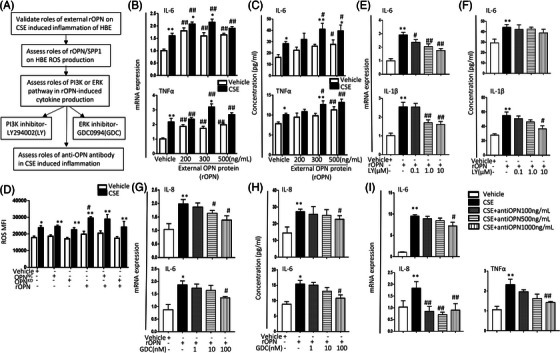
Effects of external Osteopontin (OPN) and signalling pathways in human bronchial epithelia (HBEs) inflammation. (A) Regulatory roles of *SPP1* in HBEs inflammation. (B) Levels of IL‐6 and TNFα mRNA expression in HBEs treated with 200, 300 or 500 ng/mL of external recombined human OPN protein (rOPN) for 24 h. (C) Levels of IL‐6 and TNFα protein in the supernatant of in HBEs pretreated with 200, 300 and 500 ng/mL external rOPN for 24 h. (D) ROS production in the vehicle or rOPN‐treated HBEs (*SPP1^NC^*) or *SPP1* gene (*SPP1^OE^*). (E) Levels of *IL‐6* and *IL‐1β* mRNA expression in HBEs pretreated with 0.1, 1.0 or 10 μM PI3K inhibitor LY294002 (LY) for 2 h and treated with external rOPN for 24 h. (F) Protein levels of IL‐6 and IL‐1β in the supernatant of HBEs pretreated with 0.1, 1.0 or 10 μM PI3K inhibitor LY294002 (LY) for 30 min and treated with external rOPN for 24 h. Levels of IL‐8 and IL‐6 mRNA expression (G) and secreted protein (H) in HBEs pretreated with 1.0, 10 or 100 nM ERK inhibitor GDC0994 (GDC) for 2 h and treated with external rOPN for 24 h. (I) Levels of *IL‐6*, *IL‐8* and TNFα mRNA expression in HBEs pretreated with 100, 500 or 1000 ng/mL anti‐OPN for 2 h and treated with CSE for 24 h. * and ** stand for *p* < .05 and .01, as compared with negative controls and ^#^and ^##^for *p* < .05 and .01, as compared with positive controls.

We hypothesize that secreted OPN can directly interact with epithelial cells through the autocrine from its own or the exocrine from other cells. To evaluate the direct effects of OPN proteins on epithelial inflammation characterized by alterations of inflammatory mediators, we consulted existing literature,^8^ pretreated HBEs with human recombined OPN proteins at 200, 300 and 500 ng/mL, challenged cells with vehicle or CSE, and measured mRNA expression by rtPCR, secreted proteins in the supernatant by ELISA, and intracellular proteins of inflammatory mediators by Western after cell lysing (Figure 4A). The external recombined human OPN protein (rOPN) increased mRNA expression of IL‐6 and TNFα from 200 ng/mL (Figure 4B) or levels of secreted IL‐6 and TNFα (Figure 4C) from 500 ng/mL in cells challenged with rOPN, as compared with those pretreated with vehicle (*p* < .05 or less, respectively). Levels of IL‐6 and TNFα mRNA expression or protein secretion in cells pretreated with rOPN from 200 ng/mL or from 300 ng/mL and challenged with CSE were significantly higher than those pretreated with rOPN and challenged with the vehicle. CSE challenge induced an increase in ROS production from HBEs (Figure 4D and Figure S6B), which was worsened by pretreatment with rOPN. Levels of ROS production were relatively lower in OPN*^KD^* cells pretreated with OPN and challenged with CSE.

In order to explore the effect of the OPN gene on PIK3 activation and the mechanisms involved in the modulation of the OPN gene on epithelial cell inflammation, the PI3K inhibitor LY294002 and ERK inhibitor GDC0994 were used. We found that LY294002 significantly down‐regulated rOPN‐increased expression of IL‐6 mRNA in a dose‐dependent pattern from 0.1 μM (Figure 4E), but not secreted IL‐6 protein (Figure 4F). LY294002 inhibited rOPN‐increased expression of IL‐1β mRNA from 1.0 μM and secreted IL‐1β protein at 10 μM (Figure 4E,F, *p* < .05 or less, respectively), as compared with cells pretreated with the vehicle and challenged with rOPN. GDC0994 showed inhibitory effects on rOPN‐increased expression of IL‐8 mRNA (Figure 4G) and secreted protein (Figure 4H) as well as IL‐6 mRNA (Figure 4G) and secreted protein (Figure 4H) at the high dose, as compared with cells pretreated with the vehicle and challenged with rOPN (*p* < .05, respectively). In order to evaluate the roles of secreted OPN from epithelial cells, we neutralized the secreted OPN by adding human OPN antibodies at different concentrations into the culture medium 30 min before cells were challenged with CSE. Expression of IL‐6 and TNFα mRNA in CSE‐challenged cells treated with OPN antibody at the concentration of 1000 ng/mL and IL‐8 from 100 ng/mL were significantly lower than those in CSE‐challenged cells treated with vehicle (Figure 4I).

### 3.6 Roles of OPN in intracellular signalling and production

**FIGURE 5:**
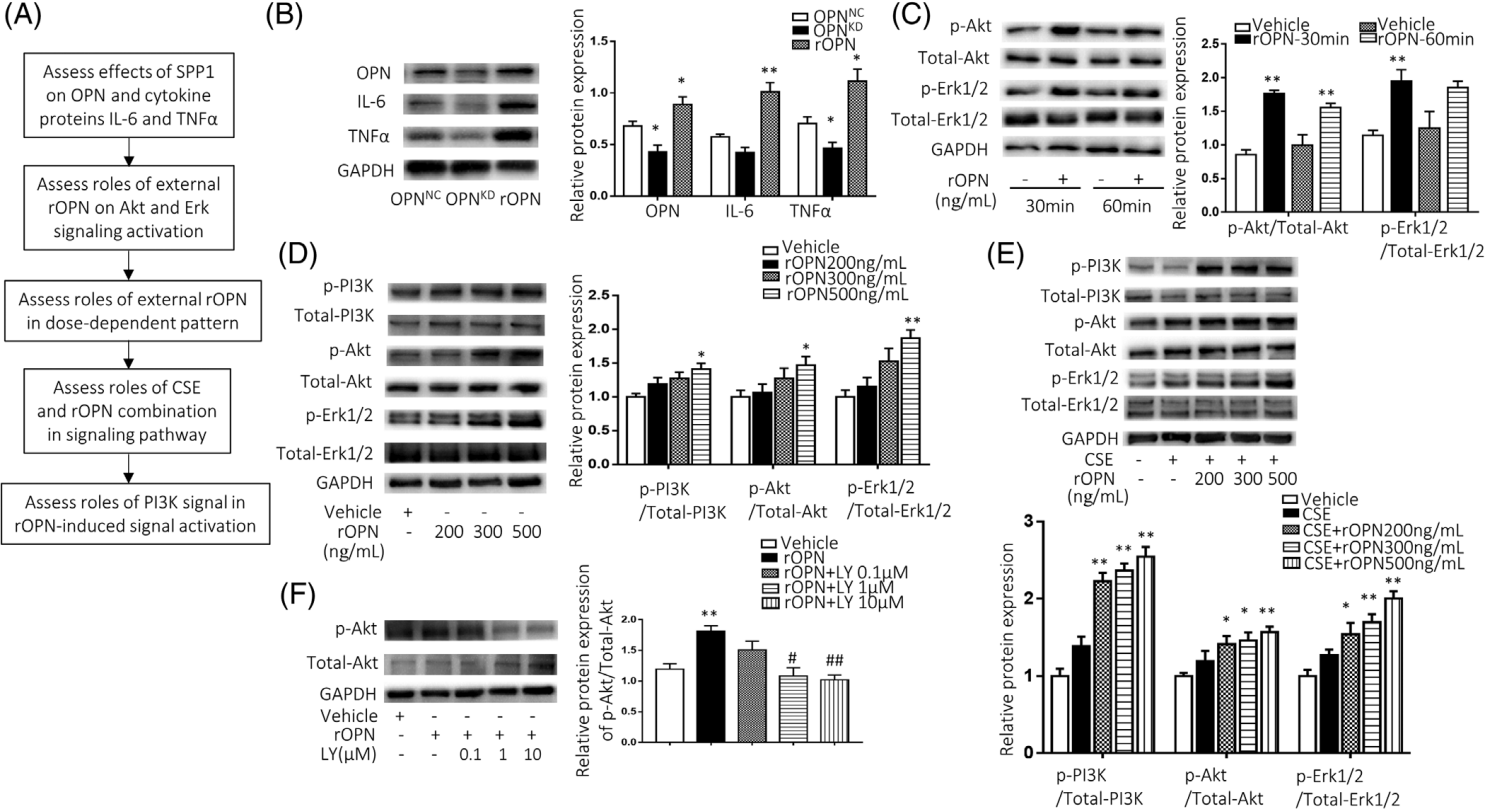
The signalling pathways mediated by Osteopontin (OPN). (A) Signalling pathways involved in regulatory roles of OPN in inflammation. (B) Levels of OPN, IL‐6 and TNFα proteins in human bronchial epithelia (HBEs) (*SPP1^NC^*) or HBEs with *SPP1 siRNA (SPP1^KD^*) treated with rOPN by western blot and quantitative analysis by ImageJ. (C) Levels of p‐Akt, Akt, p‐Erk1/2, or Erk1/2 proteins in HBEs treated with 500 ng/mL rOPN for 30 or 60 min. (D) Levels of p‐PI3K, PI3K, p‐Akt, Akt, p‐Erk1/2 or Erk1/2 proteins in HBEs treated with 200, 300 or 500 ng/mL rOPN for 24 h. (E) Levels of p‐PI3K, PI3K, p‐Akt, Akt, p‐Erk1/2 or Erk1/2 proteins in HBEs pretreated with 200, 300 or 500 ng/mL rOPN and treated for CSE for 24 h. (F) Levels of Akt and p‐Akt proteins in HBEs treated with 0.1, 1.0 or 10 μM PI3K inhibitor LY294002 (LY) for 2 h and treated with rOPN for another 24 h. * and ** stand for *p* < .05 and 0.01, as compared with negative controls and ^#^and ^##^for *p* < .05 and 0.01, as compared with positive controls.

We measured intracellular levels of OPN, IL‐6, and TNFα proteins in OPN*^NC^* or OPN*^KD^* cells as well as HBEs treated with rOPN for 24 h (Figure 5A) and found that levels of OPN, IL‐6, or TNFα were lower in OPN*^KD^* cells and higher in rOPN‐treated cells, as compared with those in OPN*^NC^* cells (Figure 5B, *p* < .05 or less). rOPN induced an increase of phosphorylated Akt and Erk proteins from 30 min and sustained to 60 min (Figure 5C, *p* < .05). rOPN‐increased levels of phosphorylated PI3K/Akt and Erk proteins appeared in a dose‐dependent pattern and significant difference at a dose of 500 ng/mL (Figure 5D). The addition of CSE at low dose increased HBEs sensitivity to external OPN, evidenced by a significant increase of phosphorylated PI3K/Akt levels at 200 ng/mL of rOPN and phosphorylated Erk protein at 200 ng/mL of rOPN (Figure 5E). Pretreatment with LY294002 (AKT phosphorylation inhibitor and mainly targeted the phosphorylation protein level^35^) down‐regulated rOPN‐increased level of phosphorylated Akt and reached statistical significance at the dose of 1 μM (Figure 5F). OPN‐*cKO* mice had lower levels of TNFα,  phosphorylated Akt, and Erk proteins after challenges with smoking (Figure S8D) or LPS (Figure S9D), as compared with WT mice.

### 3.7 Spatial distribution of OPN gene and OPN‐enriched lung epithelia

**FIGURE 6:**
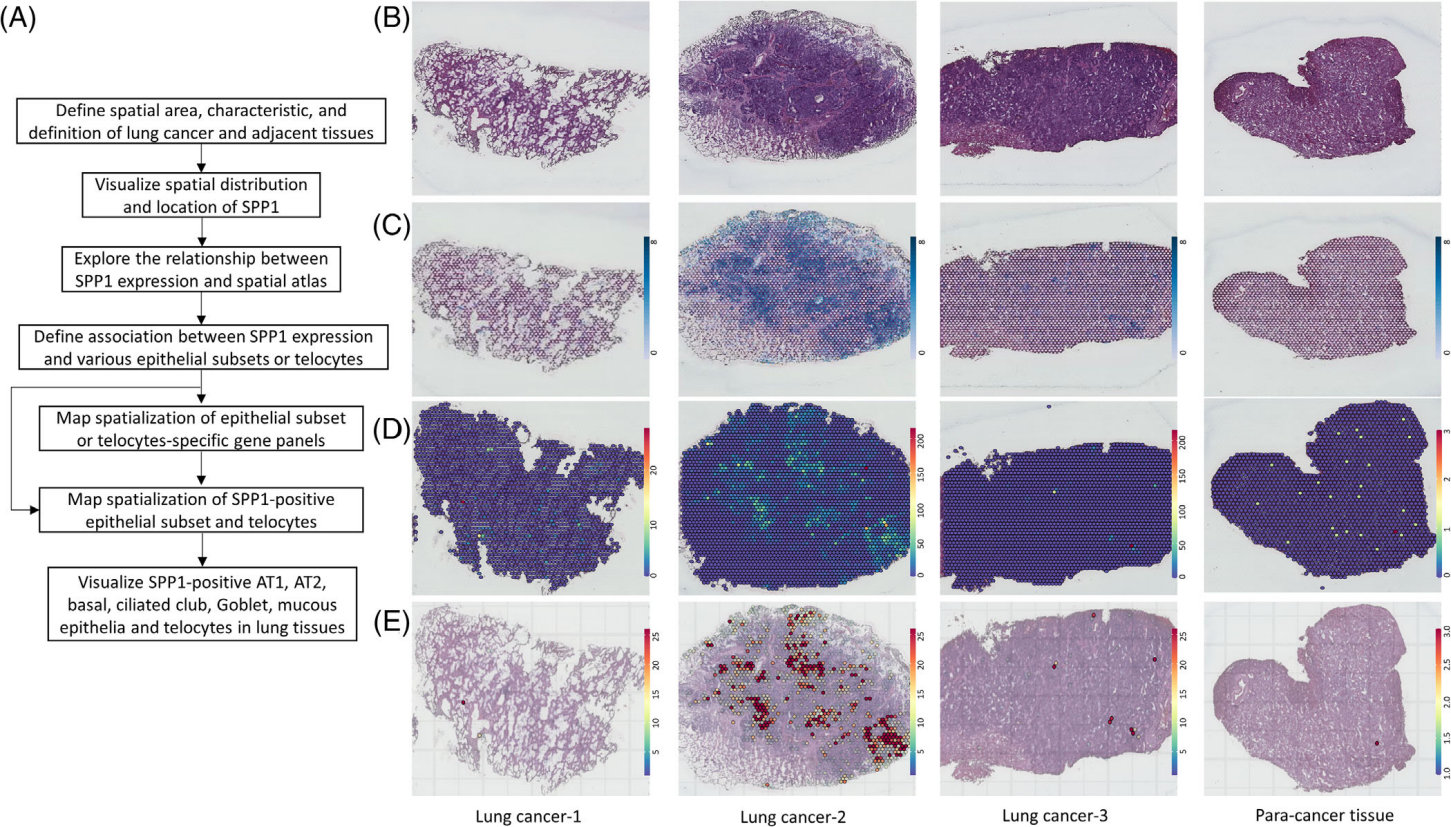
Spatial distribution of SPP1. (A) Spatialization of SPP1 and SPP1‐expressed epithelial subtypes and telocytes. (B) Spatial area of the tissue on the basis of pathological characteristics and morphological definition. (C) Spatial distribution and tissue atlas of *SPP1* gene visualized with the cell loupe browser software. (D) Distribution of *SPP1* gene expression in spatial transcriptomics spot. (E) Distribution of *SPP1* gene expression in spatial transcriptomics spot combined with HE stained tissue section.

Spatialization of SPP1 and SPP1‐expressed epithelial subtypes and TCs were analyzed and visualized using spatial transcriptomics to find out the evidence that SPP1 may play roles in the interaction between epithelial subtypes and TCs, as explained in Figure 6A. The quality sampling and procedures for spatial transcriptomics were evaluated and controlled by the combination of histological scores with RIN. Three samples were collected from patients with invasive adenocarcinoma lung cancer and one from lung squamous cell carcinoma. The size of tumour tissues was about 2–3 cm without lymph nodes and distant organ metastasis at phase I of clinical staging. Spatial areas of the tissue were selected on the basis of pathological characteristics and morphological definition, as indicated in Figure 6B. Using various analytic methodologies and imaging programs, we first defined the spatial distribution and tissue atlas of OPN gene (OPN/SPP1 sequence) and visualized the reality of OPN gene location with the cell loupe browser software (Figure 6C) and the actual difference of OPN gene expression in colour scales with Spaceranger software (Figure 6D,E). The spatial atlas demonstrated a clearly increased expression of the OPN gene in lung cancer tissues (Figure 6C,D) and the transit process from low to high expression (Figure 6E). The second sample appeared the highest expression. The position of OPN expression was relatively high in the middle of cancer tissue since the expression intensity of five regions is relatively strong. It indicates that OPN expression appears a strong localization and cell type‐dependent spatialization.

**FIGURE 7:**
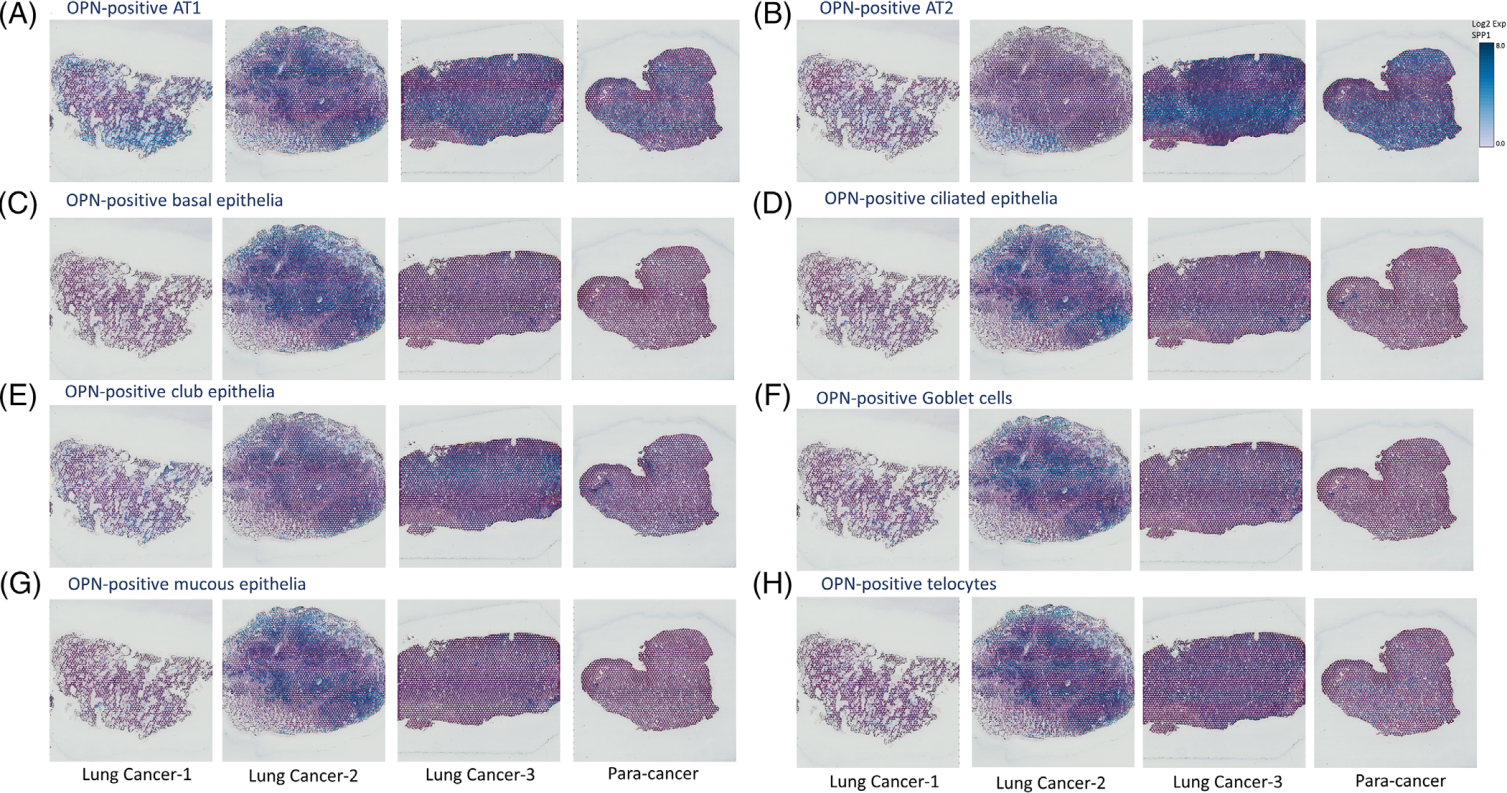
Spatial distribution of *SPP1*‐enriched lung epithelial subtypes and telocytes. Spatial distribution of *SPP1*‐positive AT1 (A), AT2 (B), basal (C), ciliated (D), club (E), Goblet (F), mucous epithelia (G) or telocytes (H) in lung cancer and para‐cancer tissues. The spots with blue colour represented the overlap expression of the *SPP1* gene and genes for lung epithelial subtypes and telocytes identification.

To understand the potential association between SPP1 expression and various epithelial subsets and TCs, we furthermore evaluated localization and spatialization of SPP1‐positive epithelial subsets by visualizing the distribution of OPN genes and epithelial subset‐specific gene panels (Figure 7). localization and spatialization of epithelial cells, endothelial cells, lymphocytes, monocytes/macrophages, stromal cells, fibroblasts and tumour cells were exhibited (Figure S10). Of those OPN‐positive epithelia, the number of OPN‐positive AT1 and AT2 were high in both lung cancer and para‐cancer tissues and the highest appearance among lung epithelial subtypes in para‐cancer tissues (Figure 7A,B). The number and density of OPN‐positive basal (Figure 7C), ciliated (Figure 7D), club (Figure 7E), Goblet (Figure 7F), and mucous epithelia (Figure 7G), as well as TCs (Figure 7H) in lung cancer tissues, were higher than those in para‐cancer lung tissues. Club epithelia and TCs had relatively higher numbers and density in para‐cancer tissues, as compared with basal, ciliated, Goblet, and mucous epithelia. The spots with blue colour represented the overlap expression of the *SPP1* gene and genes for lung epithelial subtypes and TCs identification. There was a clear association of spatial distribution between OPN‐positive epithelia and TCs. There was a clear heterogeneity of OPN‐positive cell spatialization, due to the difference of selected locations, disease severities, and clinical phenomes among lung cancer tissues.

### 3.8 Regulatory roles of OPN in cell–cell interactions

**FIGURE 8:**
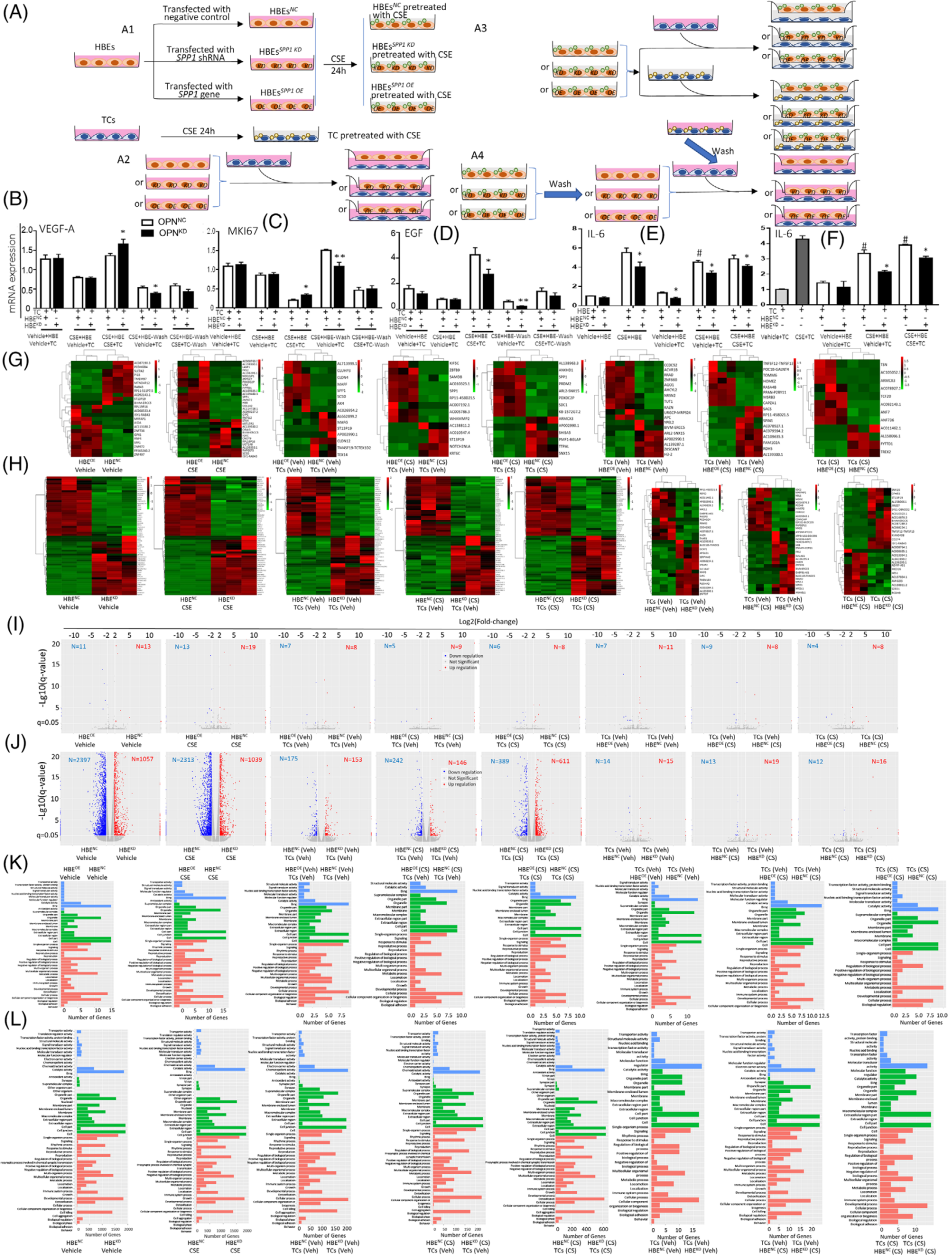
Regulatory roles of Osteopontin (OPN) in cell‐cell interactions. (A) Co‐culture study design of human bronchial epithelia (HBEs) (*SPP1^NC^*), HBEs with *SPP1 siRNA (SPP1^KD^*), HBEs with *SPP1* gene (*SPP1^OE^*), or telocytes (TCs). (A1) Study design of *SPP1^NC^*, *SPP1^KD^*, *SPP1^OE^* or TCs challenged with vehicle or CSE. (A2) HBEs (*SPP1^NC^*), HBEs with *SPP1 siRNA (SPP1^KD^*), or HBEs with *SPP1* gene (*SPP1^OE^*) co‐cultured with TCs. (A3) *SPP1^NC^*, *SPP1^KD^* or *SPP1^OE^* HBEs were challenged with CSE for 24 h and co‐cultured with TCs for another 24 h. (A4) *SPP1^NC^*, *SPP1^KD^* or *SPP1^OE^* HBEs challenged with CSE for 24 h and then washed as activated HBEs, TCs pretreated with CSE for 24 h and washed as activated TCs or activated cells cocultured for another 24 h. The mRNA levels of vascular endothelial growth factor alpha (*VEGFα*, B), *MKI67* (C), endothelial growth factor (*EGF*, D), or IL‐6 (F) in TCs and IL‐6 in HBEs (E) after co‐culturing. The heatmaps of partial up‐ (G) or down‐ (H) regulated genes of HBE*^NC^*, HBE*^SPP KD^*, HBE*^SPP1 OE^*, or TCs with statistical significance. (I) The number of different expressed genes in HBE*^SPP1 OE^* or TCs after co‐culture. (J) The number of different expressed genes in HBE*^SPP1 KD^* or TCs after the co‐culture. Cellular process, metabolic process, biological regulations or gene number related to single‐organism process or with response to stimuli between HBE*^NC^* or HBE*^SPP1 OE^* co‐cultured with TCsv (K) and between HBE*^NC^* or HBE*^SPP1 KD^* co‐cultured with TCs (L) pretreated with vehicle or CSE. * and ** stand for *p* < .05 and .01, as compared with negative controls and ^#^and ^##^for *p* < .05 and .01, as compared with positive controls.

Figure 7 displayed that the blue dots overlapped in different lung epithelial subtypes and TCs of the same sample, indicating that OPN‐positive epithelial cell subtypes might have a relationship with TCs in the spatial distribution. We further speculated that there could be an interaction between epithelia and TCs and evaluated the regulatory roles of intra‐epithelial OPN in the cell‐cell interactions between epithelial‐interstitial cells. TCs were isolated and identified by vimentin and PDGFRα, FOXL1 expression was exhibited in Figure S11. We co‐cultured TCs with OPN‐knock‐downed or OPN‐over‐expressed HBEs and challenged with vehicle or CSE, to observe whether intra‐epithelial OPN regulates the function of TCs as the representative interstitial cells (Figure 8A). The first study was designed to evaluate the regulatory effects of OPN‐down‐regulated HBEs in human TC function after challenge with vehicle or CSE (Figure 8‐A1,A2). The vascular endothelial growth factor alpha (VEGFα) and endothelial growth factor (EGF) was found as the major components within TCs.^36^ The capacity of TC proliferation was measured using *MKI67* gene expression, which codes for Ki‐67 protein and expresses in the G2 phase and mitosis.^37^, ^38^, ^39^ We found that levels of VEGFα (Figure 8B), MKI67 (Figure 8C), and EGF (Figure 8D) in TCs were significantly lower after the co‐culture with HBEs pretreated with CSE for 24 h, as compared with those with the vehicle, while there was no difference between TCs co‐cultured with OPN*^NC^* or OPN*^KD^* HBEs pretreated with vehicle or CSE. The expression of VEGFα and MKI67 mRNA significantly increased, while EGF mRNA decreased in TCs when both TCs and OPN*^KD^* HBEs were exposed to CSE, as compared with TCs and OPN*^NC^* HBEs to CSE. We noticed that the expression of VEGFα, MKI67, and EGF mRNA decreased in TCs with vehicle co‐cultured with CSE‐activated OPN*^KD^* HBEs for 24 h, as compared with CSE‐activated OPN*^NC^* HBEs. When both TCs and OPN*^KD^* HBEs or OPN*^NC^* HBEs were pre‐activated with CSE, there was no difference in mRNA expression between TCs with OPN*^KD^* HBEs or OPN*^NC^* HBEs. The expression of IL‐6 mRNA in HBEs or TCs was lower after co‐culture with CSE as compared with co‐culture with CSE‐treated HBEs or TCs alone and was significantly reduced in TCs co‐cultured with OPN*^KD^* HBEs as compared with OPN*^NC^* HBEs (Figure 8E,F).

To better understand molecular mechanisms by which OPN contributes to the epithelial‐telocyte interaction, we measured transcriptomic profiles of OPN*^NC^* HBEs, OPN*^KD^* HBEs, or OPN*^OE^* HBEs pretreated with vehicle or CSE after co‐cultured with TCs pretreated with vehicle or CSE, as well as transcriptomic profiles of TCs pretreated with vehicle or CSE after co‐cultured with OPN*^NC^* HBEs, OPN*^KD^* HBEs, or OPN*^OE^* HBEs pretreated with vehicle or CSE (Figure 8A). Figure 8G,H shows the heatmaps of partial up‐ or down‐regulated genes of OPN*^NC^* HBEs, OPN*^KD^* HBEs, OPN*^OE^* HBEs, or TCs with statistical significance. The number of DEGs in OPN*^OE^* HBEs or TCs after the interaction (Figure 8I) was much less than that in OPN*^KD^* HBEs or TCs after the interaction (Figure 8J). Gene functional categories (e.g. binding, cell part, cellular process, metabolic process, biological regulations and gene number related to single‐organism process or with response to stimuli) altered and increased between OPN*^OE^* HBEs and OPN*^NC^* HBEs co‐cultured with TCs pretreated with vehicle or CSE, while genes related with membrane functions or with organelle activities altered between TCs co‐cultured with OPN*^OE^* HBEs or OPN*^NC^* HBEs pretreated with vehicle or CSE (Figure 8K). Similar changes were also noted in the epithelial‐telocyte interaction when the OPN gene was down‐regulated (Figure 8L). Up‐regulated DEGs of OPN*^OE^*‐ (Table S4) or OPN*^KD^*‐ (Table S5) dominated epithelia‐telocyte interaction showed that some over‐expressed genes appeared between OPN*^OE^* HBEs and OPN*^NC^* HBEs challenged with vehicle or CSE, while top DEGs of HBEs or TCs in the epithelial‐telocyte interaction were different in the appearance of OPN*^OE^* HBEs and OPN*^KD^* HBEs. Similar patterns of down‐regulated DEGs were noted in OPN*^OE^*‐ (Table S6) or OPN*^KD^*‐ (Table S7) dominated epithelia‐telocyte interaction.

## 4 DISCUSSION

The present studies evidence that OPN plays an important regulatory role in epithelial origin lung inflammation and epithelial‐telocyte interaction. In addition to multiple biological functions of OPN in the cell to maintain cell shape, movement, differentiation, adherence and proliferation, OPN can be a critical factor to bridge various stages, severities, and natures of the disease. For example, OPN was proposed to link osteoarthritis and osteoporosis by regulating inflammatory activities and metabolism.^40^ Preclinical studies demonstrated that OPN contributed to inter‐organ communication as one of the cross‐talk mediators in the circulation, responsible for acute kidney injury‐induced acute lung injury.^41^ This particular study showed that kidney tubule epithelial cells generated OPN and released it into the blood to bind the receptors in the lung and initiate distant lung endothelial cell barrier dysfunction, lung tissue inflammation and oedema, and organ dysfunction. OPN is associated with inflammation,^42^, ^43^ which is consistent with our results that OPN knockdown seems to affect the basal mRNA levels of inflammatory cytokine IL8.

Gathering current findings with previous studies on blood alterations of genomic and proteomic profiles in patients with COPD, AECOPD and lung cancer,^1^, ^44^, ^45^, ^46^ the increased circulating levels of OPN proteins and expression of OPN mRNA in leukocytes are associated with chronic airway stimuli, inflammation, and cancer development, with the disease progression and combination, and with the transfer from disease stages and severities. Our data suggest that the SPP1‐centralized network among integrins, TP53, CD44 and RUNX2 play essential roles in bridging various stages of inflammation‐oriented lung diseases, evidenced by the fact that the network was consistently activated through smokers, smokers with COPD, and COPD smokers with lung cancer. OPN may contribute to the inflammation by interacting with CD44 and promoting the conformational adjustment and recruitment of additional membrane receptors like integrins.^47^

Our data on intracellular genetic modifications and external administration of OPN evidence that OPN plays a regulatory role in epithelial‐origin lung inflammation. Previous studies demonstrated that over‐expression of epithelial OPN co‐existed with the epithelial‐mesenchymal transit in lung cancer tissues.^8^ The epithelial OPN regulates lung cancer cell movement, proliferation, and EMT process by activating OPN‐PI3K and OPN‐MEK pathways and re‐organizing the conformational structure of vimentin‐associated cytoskeletons. The present study illustrated CSE‐induced lung epithelial OPN expression in a dose‐ and time‐dependent pattern. Of epithelial subtypes, OPN over‐expressed in Goblet cells of patients with COPD by single‐cell RNA sequencing, different from other lung diseases.^48^, ^49^ Our studies using the down‐/up‐regulation or genome/conditional knockout of OPN confirmed the decisive roles of intra‐ or extra‐cellular epithelial OPN in the development of epithelial lung origin inflammation. The extracellular OPN proteins, probably from epithelial autocrine or paracrine secretion, from interstitial fluid, or from the circulation by capillary leakage, can activate the neighbour and/or distant epithelial cells and other resident cells by the cell‐cell interaction. Paracrine OPN‐signaling could act as an alternative to regulating epithelial cell sensitivity to molecules in the disease.^50^ It seems that OPN is involved in the initiation of epithelial lung origin inflammation and injury, as one of the important mechanisms by which activated epithelial cells act as the secondary primer to cause acute and chronic lung injury or contribute to epithelial origin fibrogenic signals, lung repair process, and fibrotic development.^16^, ^41^, ^51^ In addition, there should be other regulatory elements that participate in epithelial lung origin inflammation and injury.^52^, ^53^

PI3K subtypes contribute to intracellular OPN signalling and vary among challenges. Extracellular OPN binds with cell surface receptors with high affinities like integrins and CD44 and activates intracellular multiple signalling pathways to achieve cell biological function, of which the PI3K signalling pathway has been suggested as one of the most important downstream signalling pathways in lung epithelial cells.^8^, ^54^ We noticed that down‐regulation of OPN resulted in the low expression of PIK3CD and PIK3C2B or high expression of PIK3C2G and PIK3R4. Of those, PIK3CD encodes p110δ to play critical roles in adaptive immunity responsible for senescent T cells, lymphadenopathy and immunodeficiency.^55^ OPN showed a clear regulatory role in PIK3CD expression in epithelial cells after being challenged with CSE or LPS. We noticed that regulatory roles of OPN in PI3K subunit expression varied among changes, for example, PIK3CA, PIK3CB, PIK3R1, PIK3R2 and PIK3R3 were more sensitive to OPN when epithelia were challenged by CSE, PIK3CD and PIK3CD when challenged with CSE or LPS, as well as PIK3CA when challenged with the combination, respectively. Our data indicates that intra‐epithelial OPN strongly signals with PI3K class I components like the catalytic part (PIK3CA/p110α, PIK3CB/p110β, PIK3CD/p110δ and PIK3CG/p110γ) and regulatory part (PIK3R1, PIK3R2 and PIK3R3) in CSE‐induced epithelial origin inflammation, although those subunits may be involved in multiple biological processes.^56^, ^57^ Our results evidenced that external OPN‐induced epithelial origin interleukin production through PI3K/ERK signal pathways, although it remains unclear how those subunits coordinate and intercommunicate in OPN‐epithelial inflammation. OPN could activate Akt/PKB and induce apoptosis by inhibiting the enzyme's ATP binding.^58^, ^59^ External OPN could increase the production of endogenous OPN, probably by inducing the nuclear translocation of the cytoplasmatic yes‐associated protein 1, upregulating transcriptional factors, stimulating JAK2/STAT3 signalling.^60^, ^61^

In addition, OPN also regulates the capacity of lung epithelial cell interactions with TCs, resulting in alterations of TC biological functions. The cell‐cell communication plays a decisive role in the maintenance of cell and organ function and microenvironmental balance.^62^, ^63^ For example, the epithelial cells interact with AT2 to generate AT2 origin inflammation through IL33, lymphatic endothelial cells intercommunicate with ST2^+^ memory (CD62L^low^CD44^hi^) CD4^+^ T cells to maintain immune function, or TCs to support survival and therapeutic effects of mesenchymal stem cells to prevent acute lung injury.^16^, ^64^ The present studies, for the first time, demonstrated the spatialization and distribution of OPN and OPN‐positive lung epithelial subtypes or TCs using spatial transcriptomics, to visualize spatial locations of OPN mRNA expression and molecular identities‐labelled cells and provide new insights for understanding molecular associations among OPN‐positive cells. It provides more comprehensive information on OPN‐positive cell neighbourhoods, co‐distributions, and potential interactions, different from the findings from in situ hybridization and immunohistochemistry to show one‐dimensional geography.^54^, ^65^, ^66^ We found that the spatial distribution varied among OPN‐positive epithelial subtypes and a close correlation between OPN‐positive epithelia and TCs.

The lung and airway TCs connect with multiple cell types to structurally support intra‐tissue cellular networks and functionally nourish connected cells through TC‐specific dendritic projectors named telopodes since they were uncovered in human lung and airway tissues.^67^, ^68^ TCs have supportive roles in the growth and survival of other cells through paralleling along with and nutritionally supporting stem cells after co‐transplantation of stem cells and TCs.^16^ Our results showed the alteration of transcriptomic profiles during the interaction between HBEs and TCs. TCs were found as part of the heterogeneity among immune cells detected by single immune cell RNA‐seq, to maintain the immune cell function through up‐regulating the expressions of adhesive molecules and receptors during the co‐culture.^15^ The present study investigated the effects of epithelial cells or OPN‐downregulated epithelial cells on TC function and showed an obvious effect of OPN‐regulated epithelial cells on TCs when both were provoked with CSE. It seems that CSE increased TC sensitivity to intercellular signals from CSE‐activated OPN‐downregulated epithelial cells. Our data emphasize the importance of CSE existence to maintain both cells under activated (or stressed) situations since such effects of OPN‐downregulated epithelial cells are lost.

To better understand molecular mechanisms by which OPN regulated epithelial interaction with TCs, we measured transcriptomic profiles of epithelial cells and TCs and found that OPN‐downregulation caused transcriptomic changes more than OPN‐upregulation in OPN‐modified epithelia and OPN‐dominated interactions between epithelia and TCs. During cell‐cell interactions, CSE induced more effects of TCs on OPN‐downregulated epithelia. Of DEGs, ZNF672 (Zinc Finger Protein 672) or BIVM‐ERCC5 (basic, immunoglobulin‐like variable motif containing an excision repair cross‐complementing rodent repair deficiency, complementation group 5 genes) was high‐ or low‐expressed in overregulated epithelia with or without CSE, while a large number in downregulated epithelia. BIVM‐ERCC5 read‐through transcript encodes a fusion protein to share sequence identity with the products of each individual gene through single‐stranded DNA binding and nuclease activity. Little has been known about the pathological significance of BIVM‐ERCC5, although its expression was proposed with cancer growth.^69^, ^70^ It seems that CSE‐activated OPN‐downregulated epithelia may change TC function and mediator production by promoting read‐through transcript activities of TCs. This can be a part of potential mechanisms by which TCs are involved in the development of lung tissue injury and repair in lung diseases.^71^ OPN‐downregulated epithelia activated with CSE mainly suppressed Protocadherin‐9 expression on TCs with or without CSE. It indicates that the member of the protocadherin family and cadherin superfamily of transmembrane proteins containing cadherin domains and networks play important roles in the epithelial‐telocyte interaction, like in other cells.^72^

In conclusion, OPN expression increased in various stages of smoke‐associated lung diseases and regulated CSE‐induced over‐production of cytokines from human bronchial epithelia evidenced by genetically modified cells and animals. CSE‐induced lung inflammation and injury reduced in animals with genome or lung conditional OPN knockout. Intra‐epithelial or external OPN strongly signal with PI3K class I components like the catalytic part and regulatory part in CSE‐induced epithelial origin inflammation. Spatial locations of OPN and OPN‐positive epithelial subtypes indicated that airway epithelia were highly associated TCs. The co‐culture experiment of epithelia and TCs further demonstrated epithelial‐telocyte cell interactions. In addition, epithelial OPN regulates epithelial communication with TCs, where down‐regulation of OPN had more effective than up‐regulation evidenced by transcriptomic profiles. Thus, the stabilization of local epithelial OPN may be an alternative for preventing and treating epithelial origin inflammation and lung injury.

## CONFLICT OF INTEREST STATEMENT

The authors declare no conflict of interest. Xiangdong Wang is an editorial board member/editor‐in‐chief for Clinical and Translational Medicine and was not involved in the editorial review or the decision to publish this article.

## Supporting information



Supporting Information
Figure S1 OPN mRNA expression from each database. *p < .05.

Supporting Information
Figure S2 Of multiple subsets of lung epithelia using scRNA‐seq, we noticed that OPN expression was significantly higher in airway goblet cells of patients with COPD, as compared with the healthy control. *p < .05.

Supporting Information
Figure S3. Expression of SPP1 in mouse chronic lung model (CS‐exposure). (A) Lung sections stained with hematoxylin and eosin and mean linear intercept (MLI). (B) Immunohistochemical (IHC) staining of OPN in mouse lung section. The expression of mRNA (C) and protein level (D) of OPN. *p < 0.05, **p < 0.01

Supporting Information
Figure S4 Expression of SPP1 in mouse acute lung model (LPS‐induced). (A) Lung sections stained with hematoxylin and eosin and lung injury score. (B) Immunohistochemical (IHC) staining of OPN in mouse lung section. The expression of mRNA (C) and protein level(D) of OPN. *p < .05, **p < .01.

Supporting Information
Figure S5 Effects of *SPP1* in lung epithelia cells inflammation. Validation efficiency of shRNA using rtPCR(A) and western blot (B). (A) Levels of *IL‐6* and *IL‐1β* mRNA in HBEs with shRNA‐3/shRNA‐4 or negative control (OPN^WT^) after CSE treated. Levels of *IL‐6* (C) and *IL‐8*(D) mRNA in A549 cells with CSE and/or LPS treated. (E) Levels of *IL‐6*, *IL‐8* and *MCP‐1* mRNA expression in A549 with negative control (A549*^NC^*) or with *SPP1*‐knockout (A549*^KO^*). mRNA expression levels of *MCP‐1* (F) in lung tissue of wild‐type mice (WT) or C57BL/6J ‐*SPP1* deficient mice (*gKO*) mice with or without LPS. Protein levels of MCP‐1 (G) in BALF WT or *gKO* mice treated with or without LPS. *^, #^ *p* < .05, **^, ##^ *p* < .01.

Supporting Information
Figure S6 ROS was measured by DCHF‐DA staining coupled with flow cytometry in the corresponding group (A, B). The apoptosis of HBE cells was assessed using flow cytometry (C). Mito‐Tracker Red CMXRos detected by flow cytometry (D).

Supporting Information
Figure S7 Schematic presentation of construction and generation of lung‐specific SPP1 knockout mice (A). The strategy of the breeding plan (B). (C) An example of PCR genotyping, For Flox: 5′‐TCTAGTTCACTGTATGGATTTTGGC‐3′, WT: one band with 153 bp; Heterozygous: two bands with 218 and 153 bp; Homozygous: one band with 218 bp. For Cre: 5′‐TGCTTCACAGGGTCGGTAG‐3′ (Cre amplicon: 210 bp). (D) Representative images of immunofluorescence staining highlighting colocalization of SPC (red), OPN (green) and DAPI (blue) in mouse lungs of WT and CKO mice.

Supporting Information
Figure S8 WT and CKO mice in the CS exposure group, lung sections stained with hematoxylin and eosin and mean linear intercept (MLI) (A). The GSH levels in the plasma (B). The TUNEL assay tests in mice lung tissue slices (C). Expression of p‐Akt, total‐Akt, p‐Erk1/2, total‐Erk1/2, TNF‐α and GAPDH in the lung were determined by Western blot(D). *n* = 5–8,*^, #^ *p* < .05, **^, ##^ *p* < .01.

Supporting Information
Figure S9 WT and CKO mice in LPS induced group, lung sections stained with hematoxylin and eosin and lung injury score (A). The GSH levels in the plasma (B). The TUNEL assay tests in mice lung tissue slices (C). Expression of p‐Akt/total‐Akt, p‐Erk1/2, total‐Erk1/2, TNF‐α and GAPDH in the lungs were determined by Western blot(D). *n* = 5–8,*^, #^ *p* < .05, **^, ##^ *p* < .01.

Supporting Information
Figure S10 Spatial distribution of *SPP1*‐enriched lung epithelial subtypes and telocytes. Spatial distribution of *SPP1*‐positive epithelial cells (A), endothelial cells (B), lymphocyte (C), monocyte/macrophage (D), stromal cells (E), fibroblast (F) and tumour cells (G) in both lung cancer and para‐cancer tissues. The spots with blue colour represented the overlap expression of the *SPP1* gene and genes for lung epithelial subtypes and telocytes identification.

Supporting Information
Figure S11 (A) Identification of primary TCs. Morphological features of telocytes from lungs. The nucleus was stained by DAPI (blue), TCs identification were Vimentin (green), FOXL1 (red) and PDGFRα (purple).

Supporting Information

## Data Availability

Data sharing is not applicable to this article as no new data were created or analyzed in this study.
