# Supplementary figures and images for: Regulatory roles of Osteopontin in lung epithelial inflammation and epithelial‐telocyte interaction
Source: Clin Transl Med. 2023 Aug 21;13(8):e1381. doi: 10.1002/ctm2.1381 (PMC10442477; doi:10.1002/ctm2.1381)

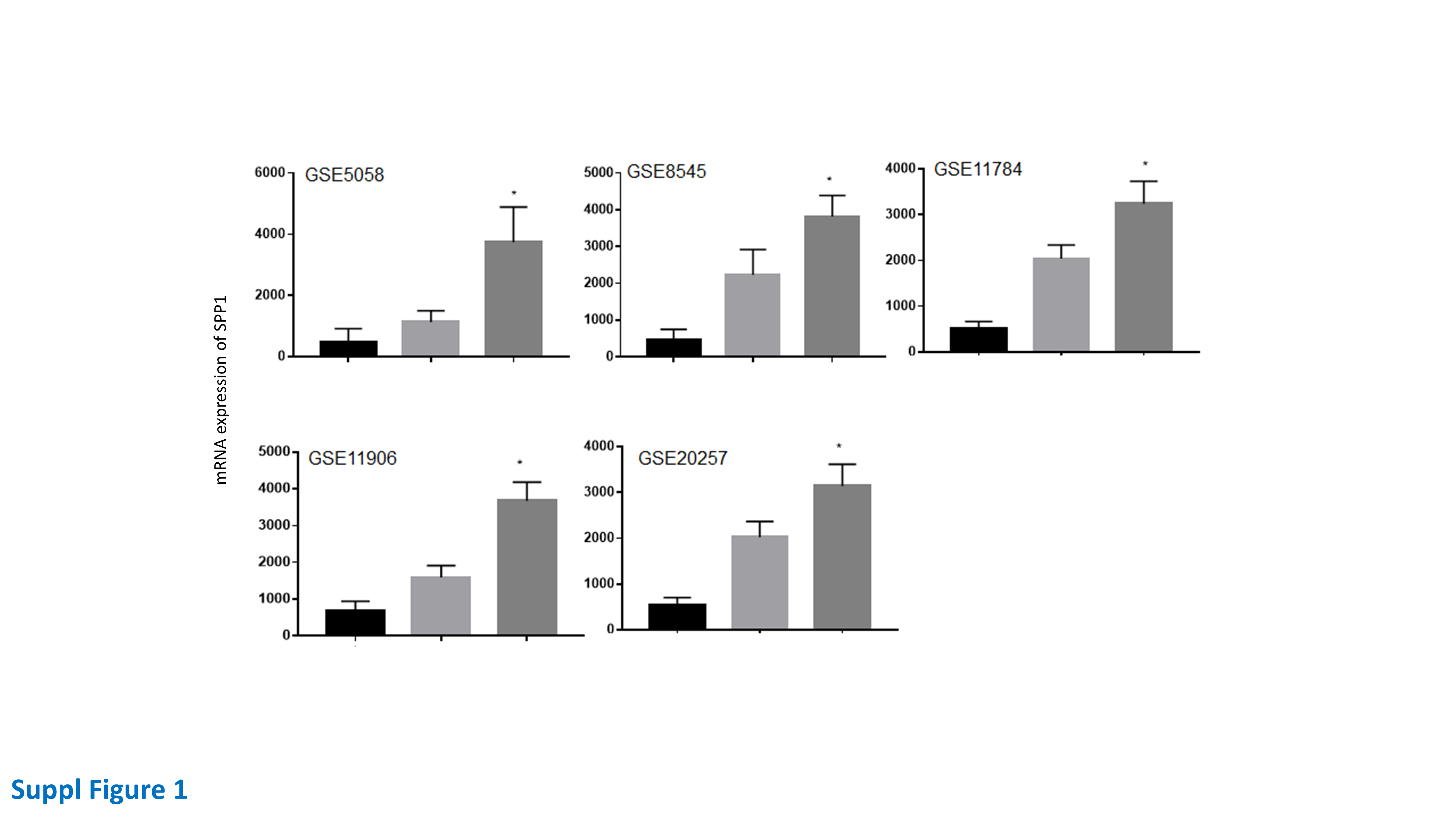

Supplement: Supplementary file 1 — Supporting Information Figure S1 OPN mRNA expression from each database. *p < .05. [file CTM2-13-e1381-s001.tif]

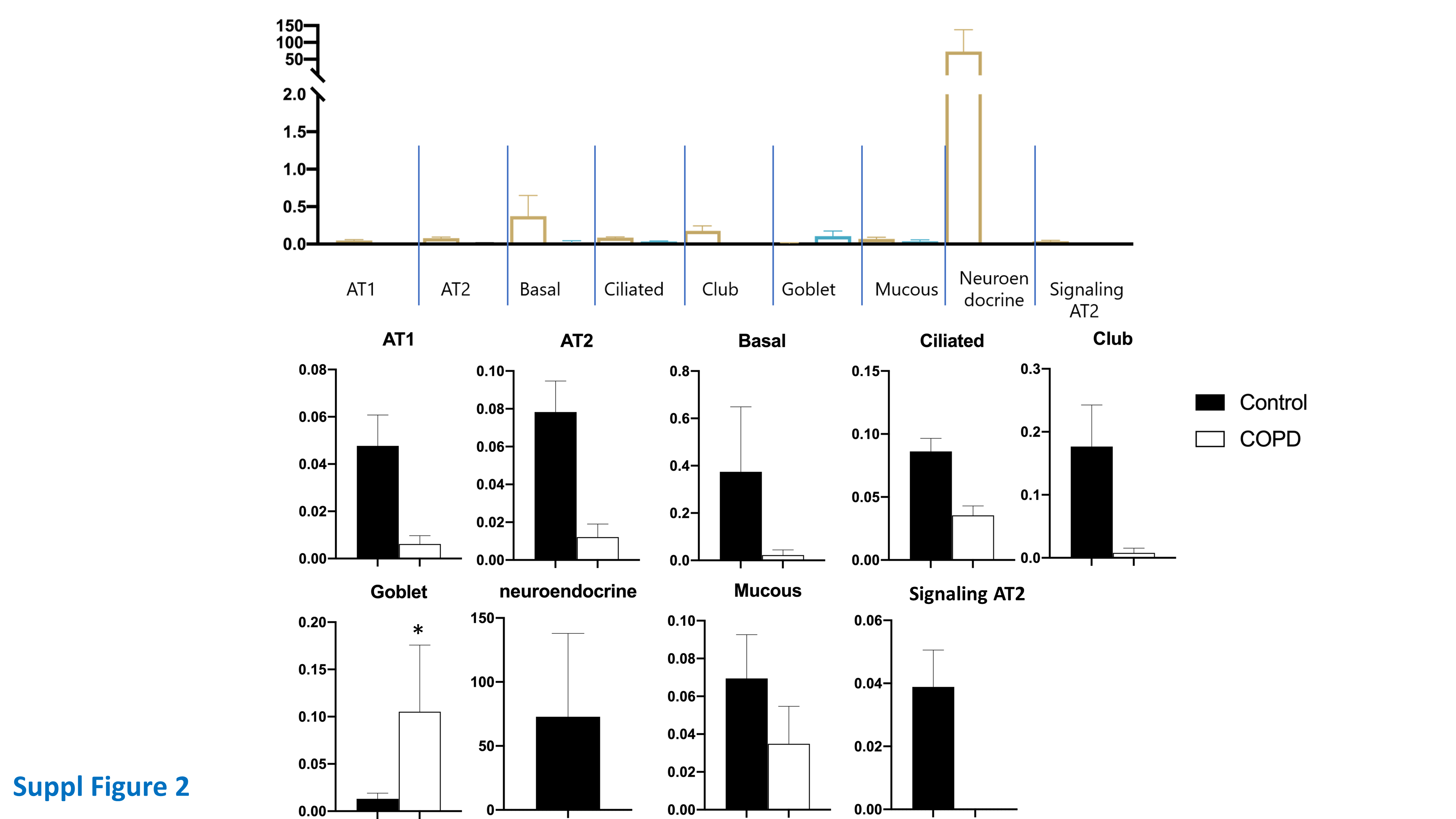

Supplement: Supplementary file 2 — Supporting Information Figure S2 Of multiple subsets of lung epithelia using scRNA‐seq, we noticed that OPN expression was significantly higher in airway goblet cells of patients with COPD, as compared with the healthy control. *p < .05. [file CTM2-13-e1381-s009.tif]

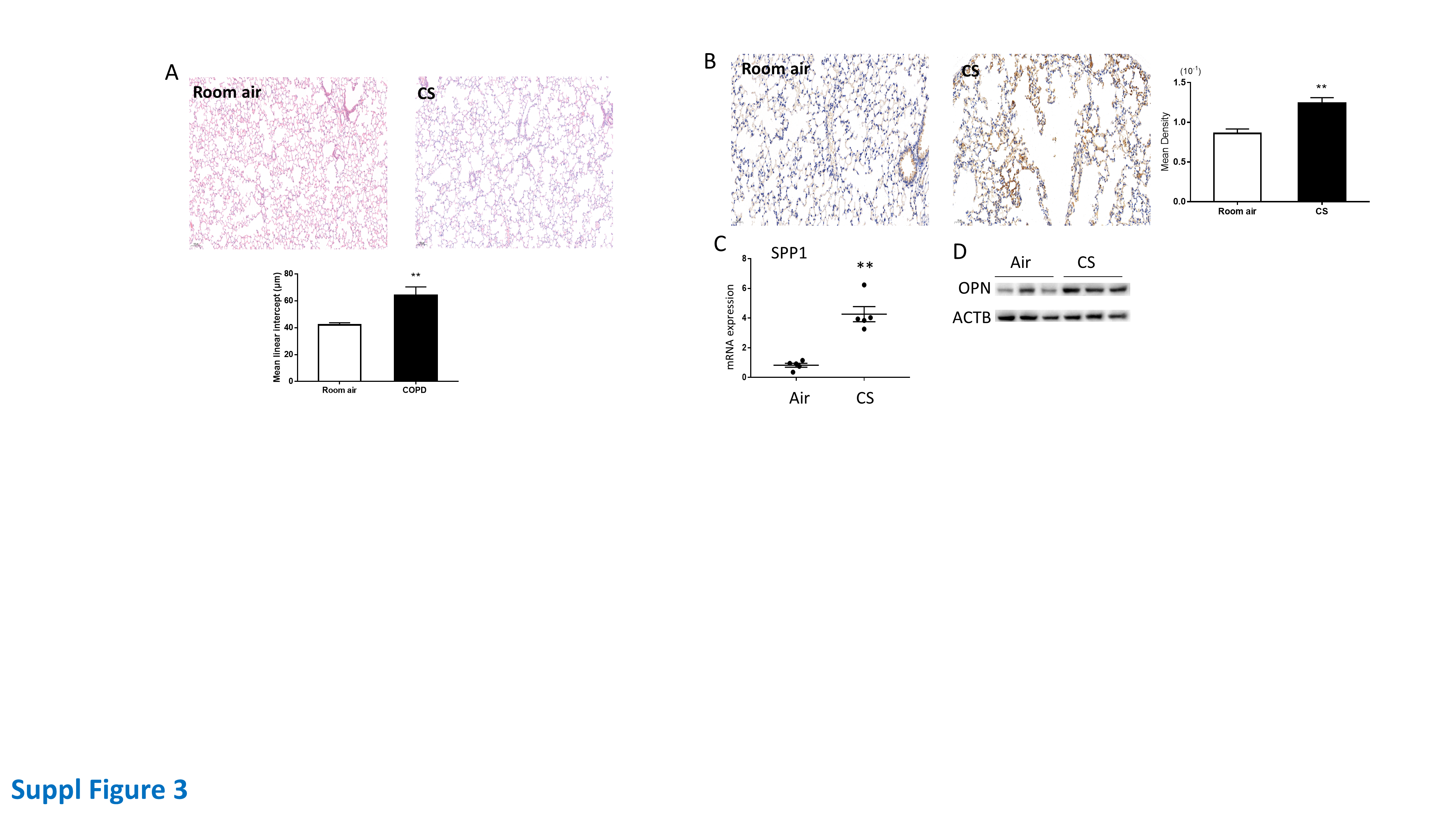

Supplement: Supplementary file 3 — Supporting Information Figure S3. Expression of SPP1 in mouse chronic lung model (CS‐exposure). (A) Lung sections stained with hematoxylin and eosin and mean linear intercept (MLI). (B) Immunohistochemical (IHC) staining of OPN in mouse lung section. The expression of mRNA (C) and protein level (D) of OPN. *p < 0.05, **p < 0.01 [file CTM2-13-e1381-s008.tif]

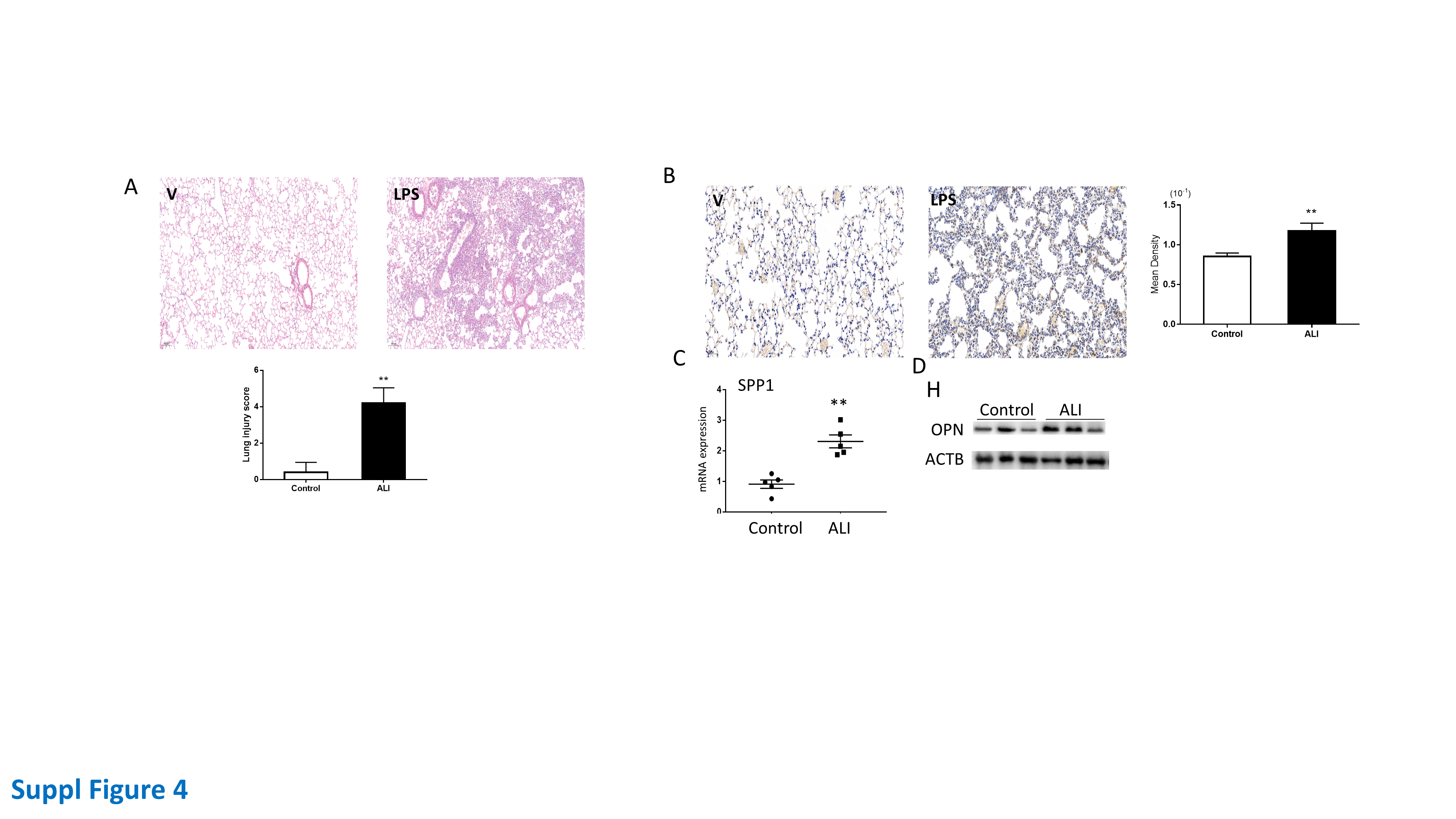

Supplement: Supplementary file 4 — Supporting Information Figure S4 Expression of SPP1 in mouse acute lung model (LPS‐induced). (A) Lung sections stained with hematoxylin and eosin and lung injury score. (B) Immunohistochemical (IHC) staining of OPN in mouse lung section. The expression of mRNA (C) and protein level(D) of OPN. *p < .05, **p < .01. [file CTM2-13-e1381-s003.tif]

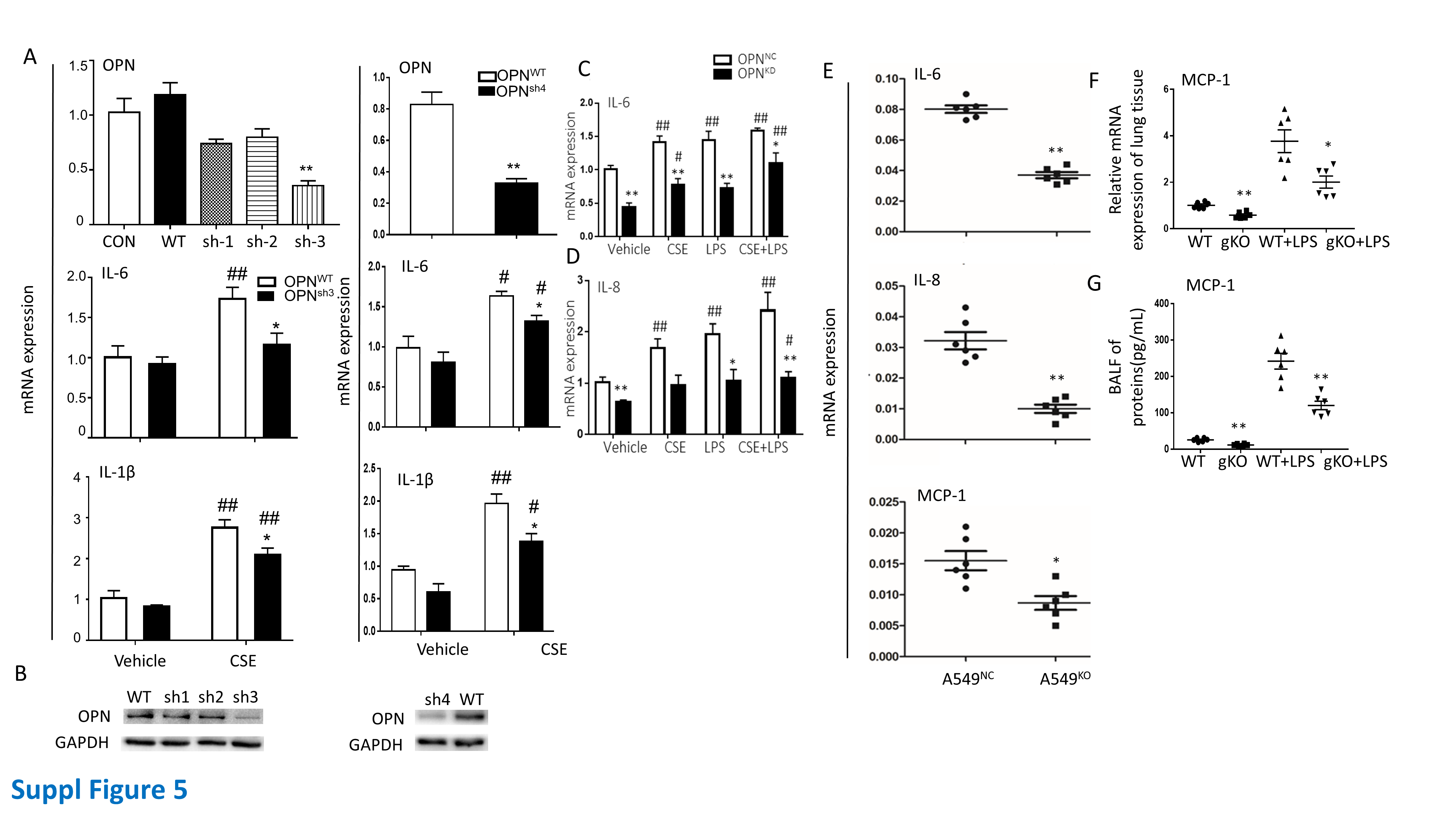

Supplement: Supplementary file 5 — Supporting Information Figure S5 Effects of SPP1 in lung epithelia cells inflammation. Validation efficiency of shRNA using rtPCR(A) and western blot (B). (A) Levels of IL‐6 and IL‐1β mRNA in HBEs with shRNA‐3/shRNA‐4 or negative control (OPNWT) after CSE treated. Levels of IL‐6 (C) and IL‐8(D) mRNA in A549 cells with CSE and/or LPS treated. (E) Levels of IL‐6, IL‐8 and MCP‐1 mRNA expression in A549 with negative control (A549 NC ) or with SPP1‐knockout (A549 KO ). mRNA expression levels of MCP‐1 (F) in lung tissue of wild‐type mice (WT) or C57BL/6J ‐SPP1 deficient mice (gKO) mice with or without LPS. Protein levels of MCP‐1 (G) in BALF WT or gKO mice treated with or without LPS. *, # p < .05, **, ## p < .01. [file CTM2-13-e1381-s002.tif]

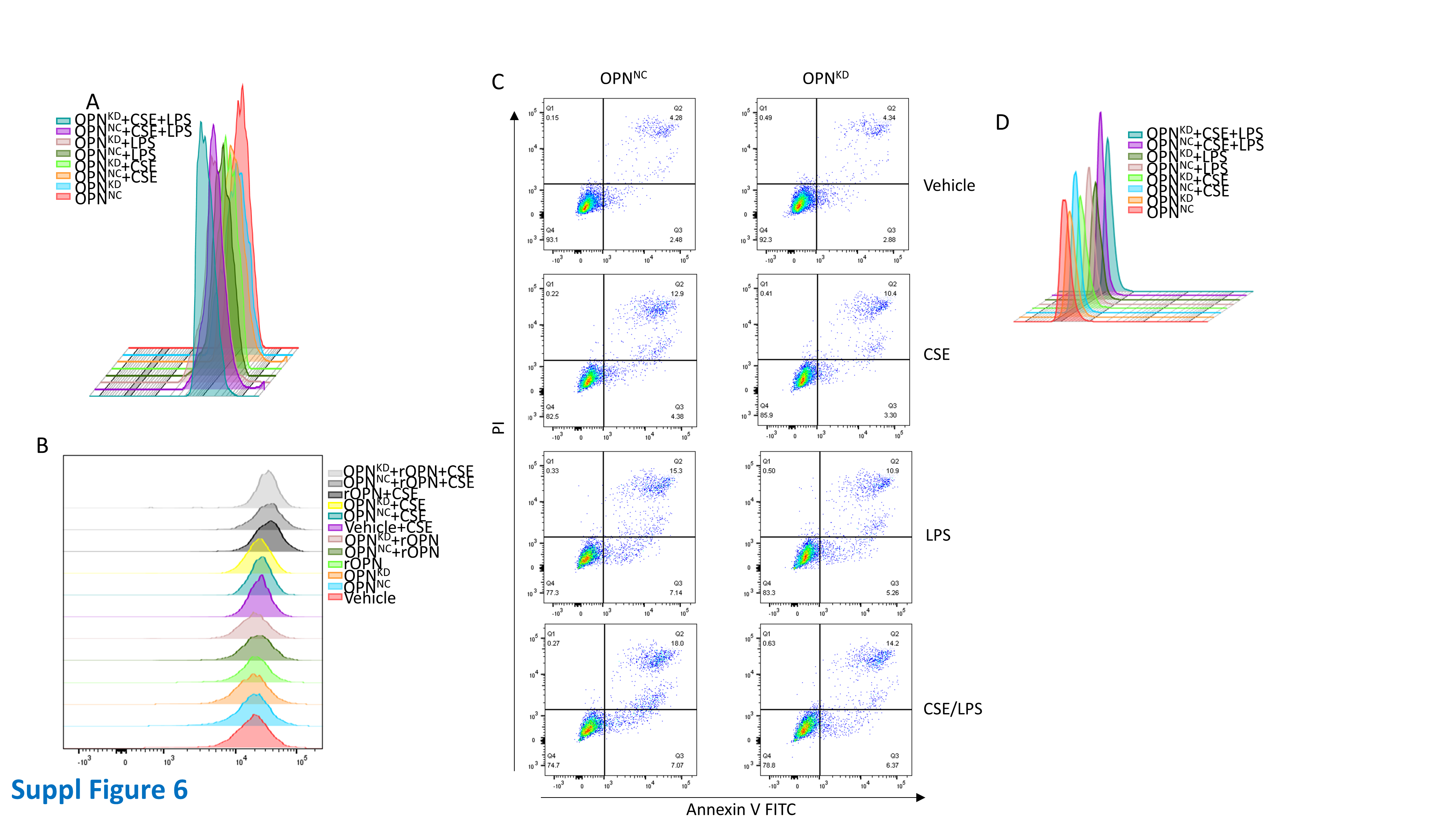

Supplement: Supplementary file 6 — Supporting Information Figure S6 ROS was measured by DCHF‐DA staining coupled with flow cytometry in the corresponding group (A, B). The apoptosis of HBE cells was assessed using flow cytometry (C). Mito‐Tracker Red CMXRos detected by flow cytometry (D). [file CTM2-13-e1381-s012.tif]

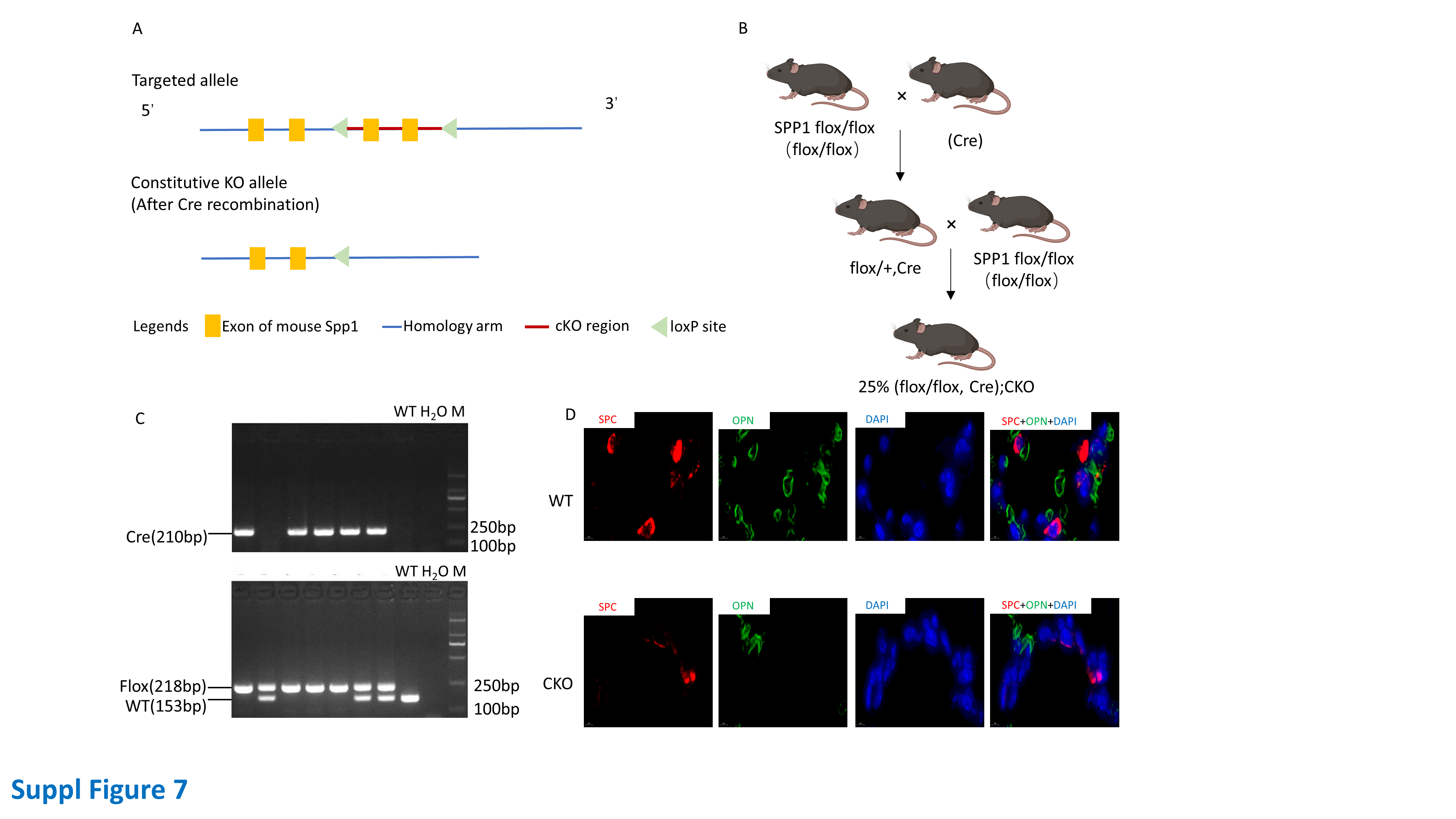

Supplement: Supplementary file 7 — Supporting Information Figure S7 Schematic presentation of construction and generation of lung‐specific SPP1 knockout mice (A). The strategy of the breeding plan (B). (C) An example of PCR genotyping, For Flox: 5′‐TCTAGTTCACTGTATGGATTTTGGC‐3′, WT: one band with 153 bp; Heterozygous: two bands with 218 and 153 bp; Homozygous: one band with 218 bp. For Cre: 5′‐TGCTTCACAGGGTCGGTAG‐3′ (Cre amplicon: 210 bp). (D) Representative images of immunofluorescence staining highlighting colocalization of SPC (red), OPN (green) and DAPI (blue) in mouse lungs of WT and CKO mice. [file CTM2-13-e1381-s006.tif]

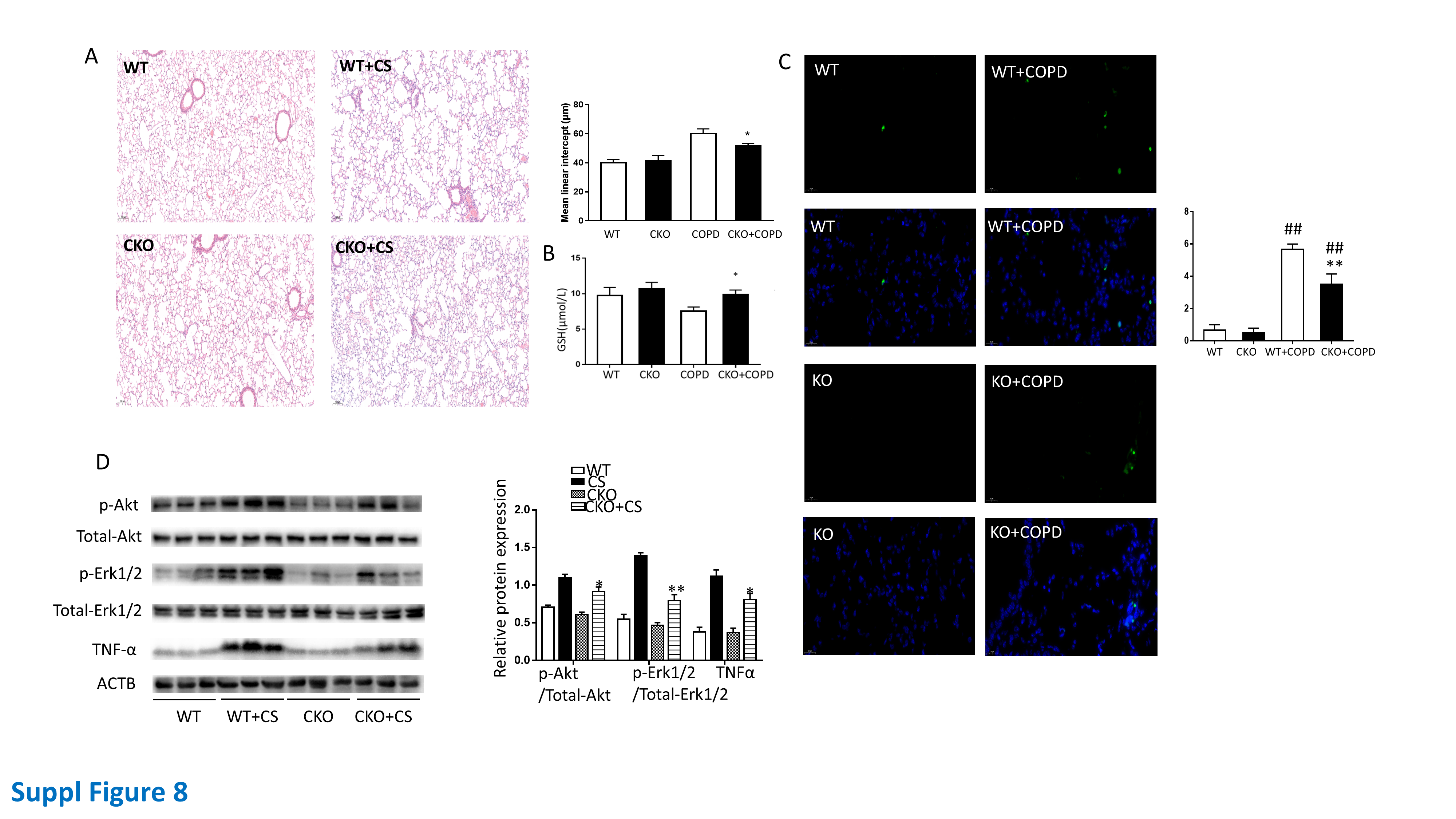

Supplement: Supplementary file 8 — Supporting Information Figure S8 WT and CKO mice in the CS exposure group, lung sections stained with hematoxylin and eosin and mean linear intercept (MLI) (A). The GSH levels in the plasma (B). The TUNEL assay tests in mice lung tissue slices (C). Expression of p‐Akt, total‐Akt, p‐Erk1/2, total‐Erk1/2, TNF‐α and GAPDH in the lung were determined by Western blot(D). n = 5–8,*, # p < .05, **, ## p < .01. [file CTM2-13-e1381-s011.tif]

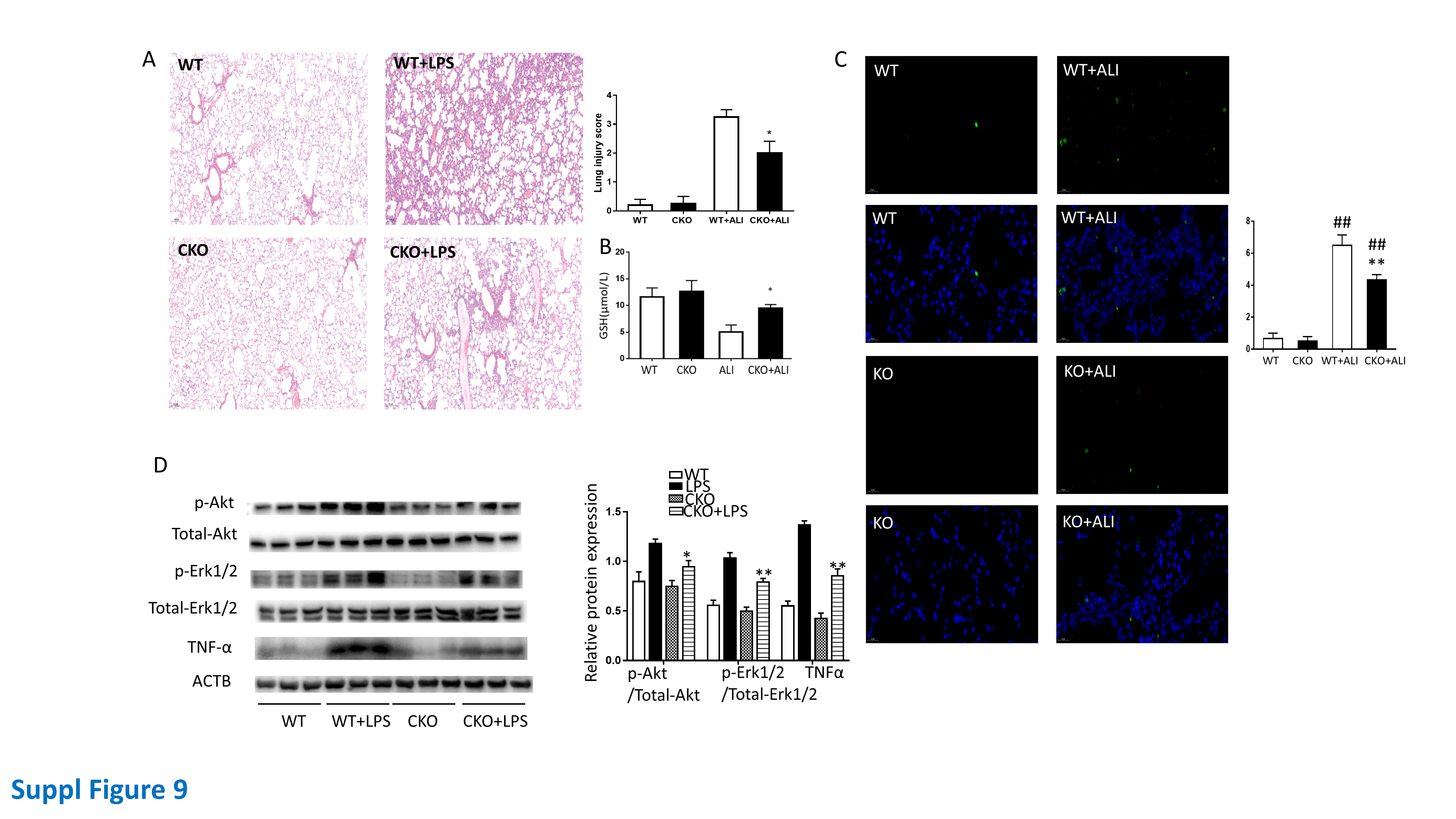

Supplement: Supplementary file 9 — Supporting Information Figure S9 WT and CKO mice in LPS induced group, lung sections stained with hematoxylin and eosin and lung injury score (A). The GSH levels in the plasma (B). The TUNEL assay tests in mice lung tissue slices (C). Expression of p‐Akt/total‐Akt, p‐Erk1/2, total‐Erk1/2, TNF‐α and GAPDH in the lungs were determined by Western blot(D). n = 5–8,*, # p < .05, **, ## p < .01. [file CTM2-13-e1381-s004.tif]

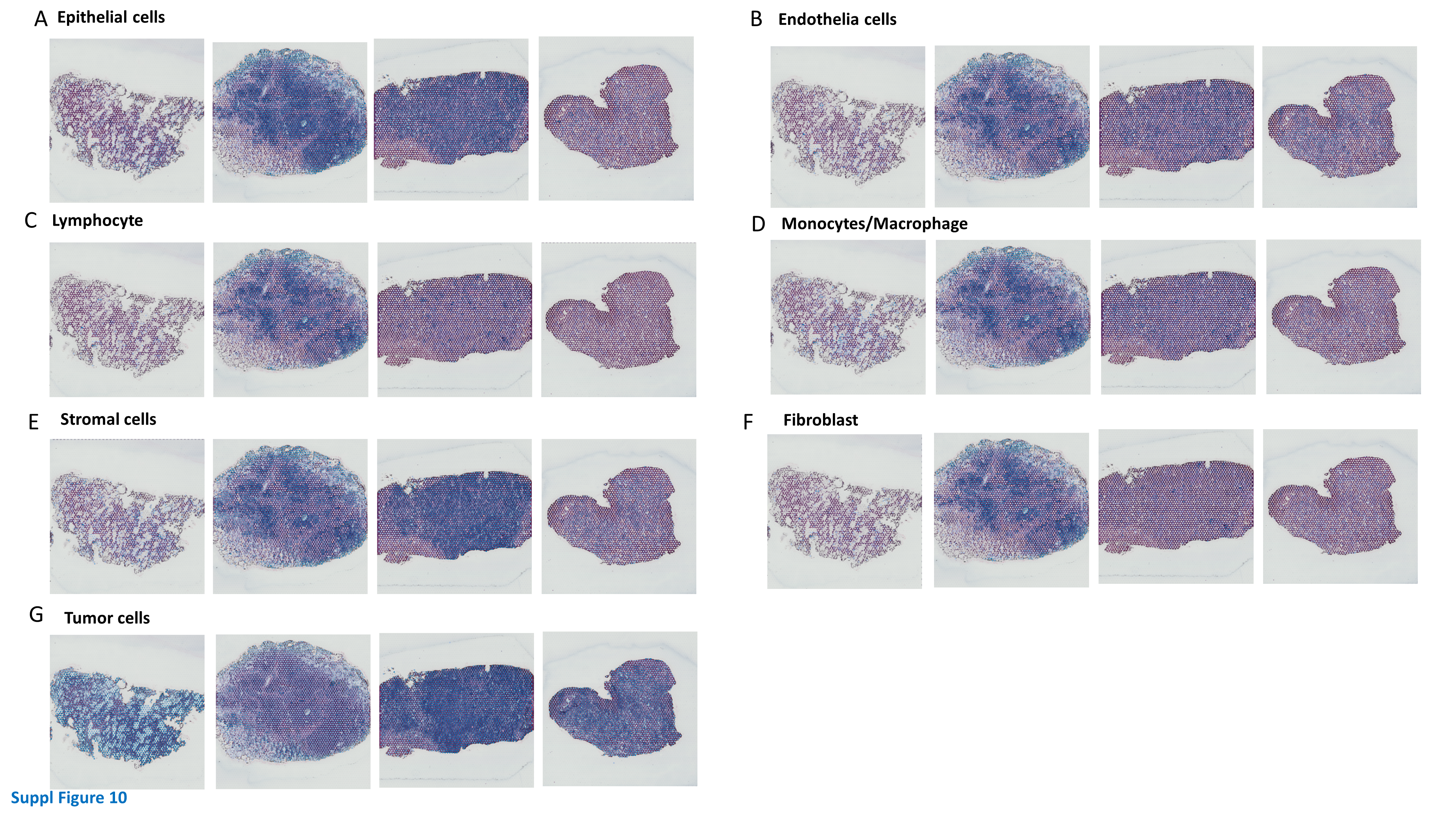

Supplement: Supplementary file 10 — Supporting Information Figure S10 Spatial distribution of SPP1‐enriched lung epithelial subtypes and telocytes. Spatial distribution of SPP1‐positive epithelial cells (A), endothelial cells (B), lymphocyte (C), monocyte/macrophage (D), stromal cells (E), fibroblast (F) and tumour cells (G) in both lung cancer and para‐cancer tissues. The spots with blue colour represented the overlap expression of the SPP1 gene and genes for lung epithelial subtypes and telocytes identification. [file CTM2-13-e1381-s005.tif]

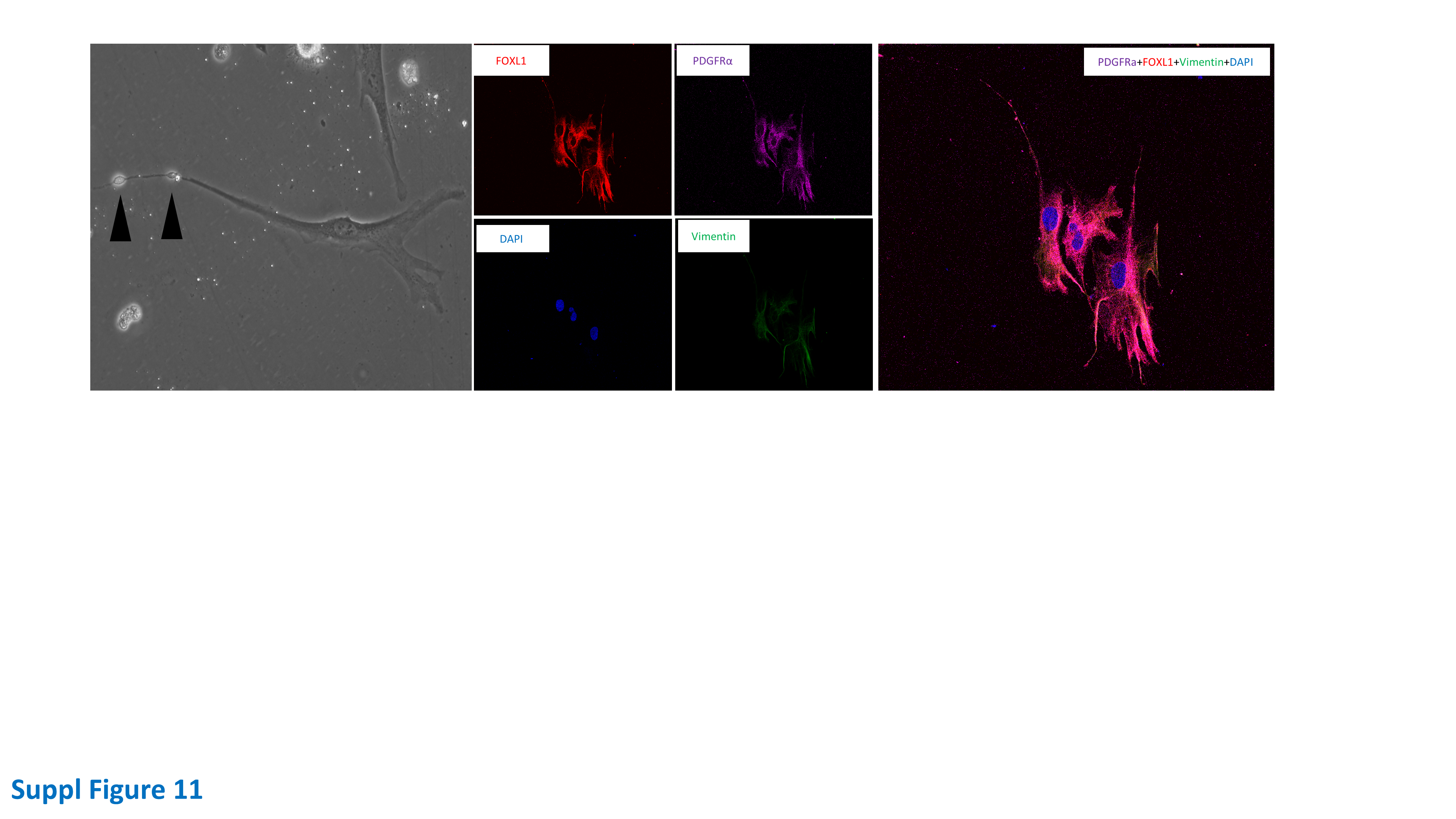

Supplement: Supplementary file 11 — Supporting Information Figure S11 (A) Identification of primary TCs. Morphological features of telocytes from lungs. The nucleus was stained by DAPI (blue), TCs identification were Vimentin (green), FOXL1 (red) and PDGFRα (purple). [file CTM2-13-e1381-s007.tif]
