# Supplementary material for: Regulatory roles of Osteopontin in lung epithelial inflammation and epithelial‐telocyte interaction
Source: Clin Transl Med. 2023 Aug 21;13(8):e1381. doi: 10.1002/ctm2.1381 (PMC10442477; doi:10.1002/ctm2.1381)
Supplement: Supplementary file 12 — Supporting Information [file CTM2-13-e1381-s010.docx]

Table 1. Antibodies used in detail.

| Antibodies | Lot number | Dilution ratio | Company |
| --- | --- | --- | --- |
| OPN | ab214050 | 1:1000 | Abcam |
| Phospho-Akt | 4060 | 1:1000 | CST |
| Akt | 4691S | 1:1000 | CST |
| Phospho-Erk1/2 | 4370S | 1:1000 | CST |
| Erk1/2 | 4696S | 1:1000 | CST |
| TNFα | 11948S | 1:1000 | CST |
| GAPDH | AF1186 | 1:1000 | Beyotime |
| HRP conjugated anti-mouse IgG | 7076 | 1:1000 | CST |
| HRP conjugated anti-rabbit IgG | 7074 | 1:1000 | CST |
| Vimentin | ab92547 | 2 µg/ml | Abcam |
| platelet-derived growth factor | 3174 | 1:1000 | CST |
| forkhead box L1 | H00002300-M09 | 1:100 | Novus |
| Prosurfactant Protein C | ab90716 | 1:100 | Abcam |

Table 2. Primers for RT-qPCR

|  | Forward | Reverse |
| --- | --- | --- |
| human |  |  |
| ACTB | AGCGAGCATCCCCCAAAGTT | GGGCACGAAGGCTCATCATT |
| SPP1 | CTCCATTGACTCGAACGACTC | CAGGTCTGCGAAACTTCTTAGAT |
| IL-6 | ACTCACCTCTTCAGAACGAATTG | CCATCTTGGAAGGTTCAGGTTG |
| IL-8 | ACTGAGAGTGATTGAGAGTGGAC | AACCCTCTGCACCCAGTTTTC |
| IL-1B | ATGATGGCTTATTACAGTGGCAA | GTCGGAGATTCGTAGCTGGA |
| TNFα | CCTCTCTCTAATCAGCCCTCTG | GAGGACCTGGGAGTAGATGAG |
| PIK3CA | CCACGACCATCATCAGGTGAA | CCTCACGGAGGCATTCTAAAGT |
| PIK3CB | TATTTGGACTTTGCGACAAGACT | TCGAACGTACTGGTCTGGATAG |
| PIK3CD | AAGGAGGAGAATCAGAGCGTT | GAAGAGCGGCTCATACTGGG |
| PIK3CG | GGCGAAACGCCCATCAAAAA | GACTCCCGTGCAGTCATCC |
| PIK3C2A | AAATGGGACCAGTAGTTTGCC | GGGTTTGTGCGGTGATTGGTA |
| PIK3C2B | TCAGGGCAATGGGGAACAC | CGTAACAGCTTGAGGTCGGTC |
| PIK3C2G | ATCAACCCCATTCTTCTAGCCA | CCAGGAGAGTTCACGGCTTTT |
| PIK3C3 | CCTGGAAGACCCAATGTTGAAG | CGGGACCATACACATCCCAT |
| PIK3R1 | ACCACTACCGGAATGAATCTCT | GGGATGTGCGGGTATATTCTTC |
| PIK3R2 | AAAGGCGGGAACAATAAGCTG | CAACGGAGCAGAAGGTGAGTG |
| PIK3R3 | TACAATACGGTGTGGAGTATGGA | TCATTGGCTTAGGTGGCTTTG |
| PIK3R4 | GCTCTTTAGGCAGTATGTGCG | GATGTCCCCATGACGAACTCC |
| ATF6 | GACAGTACCAACGCTTATGCC | CTGGCCTTTAGTGGGTGCAG |
| XBP1 | CCCTCCAGAACATCTCCCCAT | ACATGACTGGGTCCAAGTTGT |
| mouse |  |  |
| ACTB | GGCTGTATTCCCCTCCATCG | CCAGTTGGTAACAATGCCATGT |
| IL-6 | CTGCAAGAGACTTCCATCCAG | AGTGGTATAGACAGGTCTGTTGG |
| TNFα | CAGGCGGTGCCTATGTCTC | CGATCACCCCGAAGTTCAGTAG |
| SPP1 | ATCTCACCATTCGGATGAGTCT | TGTAGGGACGATTGGAGTGAAA |
| CXCL1 | ACTGCACCCAAACCGAAGTC | TGGGGACACCTTTTAGCATCTT |

Table 3 (A). Differentially expressed genes (DEGs) of non-smokers vs chronic obstructive pulmonary disease (COPD).

| Gene symbol | logFC | p-value | Gene feature |
| --- | --- | --- | --- |
| BPIFB1 | -1.294814 | 3.0307E-35 | Down |
| SCGB3A2 | 1.1924598 | 0.00022295 | Up |
| C20orf85 | -1.113695 | 2.55E-39 | Down |
| SERPINB4 | -1.087318 | 5.5142E-06 | Down |
| C9orf24 | -0.953879 | 3.0671E-28 | Down |
| AKR1B10 | -0.919112 | 0.00183832 | Down |
| UPK1B | -0.913777 | 1.9159E-08 | Down |
| SERPINB2 | -0.910074 | 5.5899E-05 | Down |
| TMEM190 | -0.891664 | 4.2083E-13 | Down |
| TCN1 | -0.875624 | 2.7239E-05 | Down |
| RSPH1 | -0.840104 | 5.5131E-23 | Down |
| FGFBP1 | -0.831929 | 1.1462E-07 | Down |
| SCGB1A1 | -0.823187 | 6.7567E-10 | Down |
| TFF3 | -0.812918 | 1.0971E-09 | Down |
| SERPINB3 | -0.807477 | 4.5248E-05 | Down |
| LRRC46 | -0.793061 | 8.271E-24 | Down |
| ALDH3A1 | -0.791828 | 1.5344E-06 | Down |
| MSMB | -0.768186 | 1.0362E-07 | Down |
| MORN5 | -0.765211 | 5.4946E-21 | Down |
| CAPSL | -0.761926 | 2.1396E-22 | Down |
| DYNLRB2 | -0.753761 | 8.3102E-20 | Down |
| C1orf194 | -0.750542 | 8.4121E-14 | Down |
| SERPINB5 | -0.742875 | 8.0102E-06 | Down |
| C9orf116 | -0.742379 | 8.5327E-26 | Down |
| ADH7 | -0.730504 | 9.9852E-10 | Down |
| GSTA1 | -0.719521 | 4.7984E-13 | Down |
| CAPS | -0.686768 | 2.7393E-16 | Down |
| ZMYND10 | -0.686013 | 1.845E-15 | Down |
| TSPAN1 | -0.685896 | 5.3444E-22 | Down |
| C11orf88 | -0.683844 | 8.7484E-13 | Down |
| DRC1 | -0.679429 | 2.2383E-15 | Down |
| DYDC2 | -0.677516 | 4.1368E-12 | Down |
| RSPH14 | -0.673139 | 5.9792E-23 | Down |
| MS4A8 | -0.665686 | 4.0781E-21 | Down |
| TPRXL | -0.660999 | 1.9149E-09 | Down |
| C11orf97 | -0.65998 | 4.2749E-14 | Down |
| DLEC1 | -0.641976 | 8.4242E-11 | Down |
| ROPN1L | -0.641573 | 6.7436E-20 | Down |
| PROK2 | -0.640478 | 0.00281028 | Down |
| FAM81B | -0.64038 | 2.5007E-13 | Down |
| AGBL2 | -0.639764 | 4.5174E-13 | Down |
| CHST6 | -0.634469 | 5.2968E-16 | Down |
| DNALI1 | -0.631643 | 5.5322E-17 | Down |
| ARMC3 | -0.628648 | 3.6133E-21 | Down |
| AKAP14 | -0.627931 | 1.6844E-14 | Down |
| TEKT2 | -0.626441 | 8.2051E-11 | Down |
| KRT23 | -0.616654 | 2.2904E-08 | Down |
| C16orf71 | -0.612423 | 8.978E-13 | Down |
| SCGB2A1 | -0.611766 | 1.7072E-07 | Down |
| SPA17 | -0.6117 | 1.9987E-20 | Down |
| MGP | 0.6107813 | 0.00094085 | Up |
| FAM229B | -0.604899 | 3.9769E-18 | Down |
| C2orf40 | -0.604836 | 2.084E-09 | Down |
| TPPP3 | -0.600698 | 1.6072E-10 | Down |
| CFAP126 | -0.599465 | 1.6042E-16 | Down |
| S100P | -0.59571 | 3.9213E-08 | Down |
| RIBC2 | -0.589687 | 3.6366E-17 | Down |

Table 3 (B). Differentially expressed genes (DEGs) of smokers vs chronic obstructive pulmonary disease (COPD).

| Gene symbol | logFC | p-value | Gene feature |
| --- | --- | --- | --- |
| CST6 | 0.755183 | 3.27E-20 | Up |
| PROS1 | -0.75251 | 1.08E-17 | Down |
| TCN1 | 1.079556 | 6.13E-15 | Up |
| CCL2 | 0.794448 | 3.03E-14 | Up |
| CEACAM5 | 0.930284 | 4.08E-14 | Up |
| FGFBP1 | 0.722976 | 2.93E-13 | Up |
| MMP12 | 0.743089 | 1.47E-12 | Up |
| DEFB1 | 0.602533 | 3.53E-12 | Up |
| DDX17 | -0.61649 | 4.38E-12 | Down |
| AQP9 | 0.677496 | 1E-10 | Up |
| SPP1 | 1.199316 | 1.74E-10 | Up |
| ELMOD1 | 0.616648 | 3.62E-10 | Up |
| PROK2 | 0.685242 | 4.19E-10 | Up |
| UPK1B | 0.692626 | 9E-10 | Up |
| BCL2A1 | 0.761278 | 2.99E-09 | Up |
| CD163 | 0.693342 | 3.36E-09 | Up |
| LTF | -0.75325 | 3.49E-09 | Down |
| C3 | -0.6742 | 8.61E-09 | Down |
| CYP1B1 | 1.083915 | 1.06E-08 | Up |
| TFF1 | 0.739301 | 1.21E-08 | Up |
| FPR1 | 0.622438 | 3.05E-08 | Up |
| ALOX5AP | 0.588577 | 2.84E-07 | Up |
| MGP | -0.58949 | 3.21E-07 | Down |
| SCGB3A2 | -1.0153 | 5.51E-07 | Down |
| VSIG4 | 0.630787 | 5.82E-07 | Up |
| SPRR3 | 0.761239 | 6.87E-07 | Up |
| MCEMP1 | 0.620795 | 1.66E-06 | Up |
| CPA3 | 0.600029 | 1.74E-06 | Up |
| CYP1A1 | 0.994611 | 2.34E-06 | Up |
| KRT6A | 0.589857 | 1.95E-05 | Up |
| C15orf48 | 0.585855 | 4.5E-05 | Up |
| AKR1B10 | 0.785846 | 5.4E-05 | Up |
| UCHL1 | 0.632317 | 0.000348 | Up |
| SFTPA2 | 0.617621 | 0.001474 | Up |

Table 3 (C). Differentially expressed genes (DEGs) of chronic obstructive pulmonary disease (COPD) vs squamous cell carcinoma (SCC).

| Gene symbol | logFC | p-value | Gene feature |
| --- | --- | --- | --- |
| BPIFA1 | -4.47025 | 6.45E-13 | Down |
| BPIFB1 | -4.37625 | 3.8E-104 | Down |
| SCGB3A1 | -4.30231 | 9.01E-34 | Down |
| AKR1B10 | 4.051232 | 2.07E-21 | Up |
| C20orf85 | -4.04306 | 4.4E-111 | Down |
| C9orf24 | -3.62243 | 1.39E-87 | Down |
| RSPH1 | -3.45386 | 4.93E-79 | Down |
| CYP4B1 | -3.44375 | 1.07E-51 | Down |
| TMEM190 | -3.17586 | 9.89E-50 | Down |
| UCHL1 | 3.16988 | 3.21E-13 | Up |
| CAPS | -3.16904 | 6.04E-72 | Down |
| C11orf88 | -3.11352 | 2.96E-55 | Down |
| SCGB1A1 | -3.11257 | 5.56E-41 | Down |
| C1orf194 | -3.08454 | 7.76E-55 | Down |
| MSMB | -3.07685 | 1.78E-47 | Down |
| DYNLRB2 | -3.00857 | 4.92E-72 | Down |
| LCN2 | -2.9584 | 4.32E-35 | Down |
| DNALI1 | -2.92844 | 3.6E-76 | Down |
| LRRC46 | -2.84531 | 7.79E-72 | Down |
| PIGR | -2.81108 | 2.93E-38 | Down |
| CAPSL | -2.80723 | 5.03E-70 | Down |
| C9orf116 | -2.76 | 1.55E-76 | Down |
| SLPI | -2.73765 | 9.39E-58 | Down |
| ZMYND10 | -2.73746 | 2.22E-56 | Down |
| AGR3 | -2.6936 | 5.52E-52 | Down |
| TSPAN1 | -2.68995 | 1.6E-77 | Down |
| MORN5 | -2.64265 | 1.97E-67 | Down |
| SPRR1B | 2.639907 | 1.63E-14 | Up |
| KRT6B | 2.620875 | 1.25E-27 | Up |
| SERPINB4 | -2.6192 | 8.56E-12 | Down |
| FAM81B | -2.61812 | 2.2E-58 | Down |
| TFF3 | -2.61758 | 1.43E-43 | Down |
| AGR2 | -2.61395 | 4.68E-59 | Down |
| HBB | -2.61226 | 5.41E-11 | Down |
| MS4A8 | -2.61108 | 1.24E-76 | Down |
| PLEKHS1 | -2.6067 | 1.03E-48 | Down |
| TMC5 | -2.60387 | 2.12E-50 | Down |
| RRAD | -2.55672 | 3.92E-55 | Down |
| CFAP70 | -2.5456 | 1.98E-40 | Down |
| S100A8 | 2.541846 | 2.51E-10 | Up |
| S100P | -2.53664 | 8.28E-50 | Down |
| MUC16 | -2.51897 | 2.29E-43 | Down |
| TEKT2 | -2.51517 | 5.6E-46 | Down |
| C11orf97 | -2.50776 | 1.63E-53 | Down |
| CRIP1 | -2.50493 | 4E-34 | Down |
| ARMC3 | -2.495 | 1.13E-70 | Down |
| ROPN1L | -2.48094 | 2E-72 | Down |
| AGBL2 | -2.46726 | 8.63E-48 | Down |
| C2orf40 | -2.42402 | 3.94E-47 | Down |
| GPX2 | 2.42063 | 5.67E-25 | Up |
| VMO1 | -2.41564 | 2.15E-23 | Down |
| TPPP3 | -2.40173 | 1.46E-46 | Down |
| KRT6C | 2.392293 | 3.69E-12 | Up |
| AKAP14 | -2.38291 | 1.1E-55 | Down |
| DRC1 | -2.3809 | 6.16E-48 | Down |
| SERPINB3 | -2.37981 | 3.67E-13 | Down |
| DYDC2 | -2.37596 | 9.52E-46 | Down |
| TSPAN8 | -2.37342 | 2.35E-47 | Down |
| OSCP1 | -2.36953 | 5.01E-70 | Down |
| PIFO | -2.36888 | 3.54E-63 | Down |
| CFAP52 | -2.30596 | 5.04E-51 | Down |
| SLC44A4 | -2.30509 | 3.4E-39 | Down |
| KRT6A | 2.304331 | 3.3E-08 | Up |
| CFAP126 | -2.27866 | 9.83E-67 | Down |
| VSTM2L | -2.27819 | 2.79E-39 | Down |
| PRR29 | -2.24527 | 6.11E-55 | Down |
| SMIM22 | -2.22728 | 4.67E-68 | Down |
| DNAH10 | -2.22552 | 3.8E-70 | Down |
| C16orf71 | -2.20968 | 1.14E-43 | Down |
| CCDC146 | -2.20205 | 1.74E-43 | Down |
| FAM229B | -2.17829 | 2.25E-60 | Down |
| CATSPERD | -2.17784 | 3.79E-30 | Down |
| ZBBX | -2.17431 | 2.93E-37 | Down |
| C9orf135 | -2.17336 | 1.18E-39 | Down |
| PSCA | -2.15849 | 2.01E-09 | Down |
| SPP1 | 2.154849 | 3.98E-10 | Up |
| CXCL1 | -2.13526 | 1.18E-32 | Down |
| RSPH14 | -2.13046 | 3.63E-60 | Down |
| NME5 | -2.12498 | 2.65E-51 | Down |
| DSG3 | 2.124777 | 1.37E-31 | Up |
| SCGB2A1 | -2.10571 | 3.71E-31 | Down |
| DNAI1 | -2.10546 | 2.12E-47 | Down |
| SPRR3 | 2.104405 | 1.02E-07 | Up |
| CXCL17 | -2.09348 | 5.87E-43 | Down |
| C1orf87 | -2.07868 | 1.48E-52 | Down |
| DLEC1 | -2.06946 | 1.65E-27 | Down |
| MNS1 | -2.0589 | 1.8E-40 | Down |
| FOLR1 | -2.05304 | 1.78E-18 | Down |
| CRYM | -2.0327 | 2.41E-30 | Down |
| FANK1 | -2.03107 | 6.43E-48 | Down |
| RFX2 | -2.01977 | 4.44E-53 | Down |
| FOSB | -2.01946 | 1.04E-13 | Down |
| FBXO15 | -2.01097 | 9.3E-45 | Down |
| LRRC34 | -1.99963 | 6.56E-31 | Down |
| EFCAB1 | -1.99796 | 3.89E-50 | Down |
| SPAG17 | -1.99535 | 3.52E-37 | Down |
| PIH1D3 | -1.98568 | 2.64E-40 | Down |
| AQP5 | -1.984 | 5.63E-25 | Down |
| TTC25 | -1.97191 | 8.08E-40 | Down |
| FOXJ1 | -1.96413 | 7.1E-35 | Down |

Table 4. Up-regulation differentially expressed genes (DEGs) resulted from the RNA-seq data

| HBEs*^SPP1 OE^*(Veh)  vs HBEs*^NC^*(Veh) | HBEs^OE^ (CSE)  vs HBEs^NC^(CSE) | HBEs^OE^/TCs(Veh)  vs HBEs^NC^/TCs(Veh) | HBEs^OE^ (CSE)/TCs (Veh)  vs HBEs^NC^ (CSE)/TCs (Veh) | HBEs^OE^(CSE)/TCs(CSE)  vs HBEs^NC^(CSE)/TCs(CSE) | TCs(Veh)/HBEs^OE^(Veh)  vs TCs(Veh)/HBEs^NC^(Veh) | TCs(Veh)/HBEs^OE^(CSE)  vs TCs(Veh)/HBEs^NC^(CSE) | TCs(CSE)/HBEs^OE^(CSE)  vs TCs(CSE)/HBEs^NC^(CSE) |
| --- | --- | --- | --- | --- | --- | --- | --- |
| SPP1  AC113188.2  ZNF497  ZNF672  RNF4  GPX1  MRFAP1  ZNF736  AIDA  FP565260.2  RPL19P16  AC068533.4  ISY1-RAB43 | SPP1  PDXDC2P  NOC2LP1  CIPC  VIM  ZNF672  LASP1  ZNF527  FRS2  RN7SL2  FYTTD1  AC114728.1  AC109460.3  AL136295.1  COL1A2  AC090227.2  AC008763.3  AL139300.1  AC109635.3 | CLDN4  SPP1  AC026954.2  AK4  MAFF  CLUHP3  SC5D  AL713999.1 | SPP1  RP11-458D21.5  WHAMMP2  SAMD8  AC010323.1  AC007192.1  AC005786.3  ZBTB9  KIF5C | SPP1  PDXDC2P  KB-1572G7.2  ARL2-SNX15  PRDM2  ANKHD1  SDC1  AL138963.3 | ZNF660  APC  ACVR1B  AGO1  KAZN  TUT1  CCDC92  NRSN2  RRAD  AHCYL2  URGCP-MRPS24 | POC1B-GALNT4  TOMM6  RASA4B  MSRB3  HOMEZ  CAPZA1  PPAN-P2RY11  TNFSF12-TNFSF13 | AC105052.3  AC078927.1  TCF20  ZNF7  TSN  ZNF736  ARMCX3  AC092143.1 |

HBEs*^NC^* treated with vehicle and HBEs*^SPP1 OE^* treated with vehicle [HBEs^OE^ (Veh) vs HBEs *^NC^* (Veh) ];

HBEs*^NC^* treated with CSE and HBEs*^SPP1 OE^* treated with CSE [HBEs^OE^ (CSE)vs HBEs *^NC^*(CSE) ];

HBEs*^NC^* co-culture with TCs with vehicle and HBEs*^SPP1 OE^* co-culture with TCs with vehicle [HBEs ^OE^/TC(Veh) vs HBEs ^NC^/TC(Veh)];

HBEs*^NC^* pretreated with CSE co-culture with TCs and HBEs *^SPP1 OE^* pretreated with CSE co-culture with TCs [HBEs^OE^ (CSE)/TCs (Veh) vs HBEs^NC^ (CSE)/TCs (Veh)];

HBEs*^NC^* co-culture with TCs with CSE and HBEs *^SPP1 OE^* co-culture with TCs with CSE [HBEs ^OE^(CSE)/TCs(CSE) vs HBEs ^NC^(CSE)/TCs(CSE)];

TCs co-culture with HBEs*^NC^* with vehicle and TCs co-culture with HBEs*^SPP1 OE^* with vehicle [TCs(Veh)/HBEs^OE^(Veh) vs TCs(Veh)/HBEs^NC^(Veh)];

TCs co-culture with HBEs*^NC^* pretreated with CSE and TCs co-culture with HBEs *^SPP1 OE^* pretreated with CSE [TCs(Veh)/HBEs^OE^(CSE) vs TCs(Veh)/HBEs^NC^(CSE)]

TCs co-culture with HBEs*^NC^* with CSE and TCs co-culture with HBEs *^SPP1 OE^* with CSE [TCs(CSE)/HBEs^OE^(CSE) vs TCs(CSE)/HBEs^NC^(CSE)].

Table 5. Up-regulation differentially expressed genes (DEGs) resulted from the RNA-seq data

| HBEs^KD^(Veh)  vs HBEs^NC^(Veh) | HBEs^KD^(CSE)  vs HBEs^NC^(CSE) | HBEs^KD^/TC(Veh)  vs HBEs^NC^/TC(Veh) | HBEs^KD^ (CSE)/TC (Veh)  vs HBEs^NC^ (CSE)/TC (Veh) | HBEs^KD^(CSE)/TCs(CSE)  vs HBEs^NC^(CSE)/TCs(CSE) | TCs(Veh)/HBEs^KD^(Veh)  vs TCs(Veh)/HBEs^NC^(Veh) | TCs(Veh)/HBEs^KD^(CSE)  vs TCs(Veh)/HBEs^NC^(CSE) | TCs(CSE)/HBEs^KD^(CSE)  vs TCs(CSE)/HBEs^NC^(CSE) |
| --- | --- | --- | --- | --- | --- | --- | --- |
| POSTN  AC010132.3  LINC02434  IL1RL1  TFF3  MYH16  IGF2BP2-AS1  CST1  LINC00460  INHBA  CSF2  ZNF763  RFPL4A  LINC02742  AC039056.2  TCN1  RNF212B  KPNA7  OLAH  SLIT1  SLC22A1  IL31RA  CLDN4  AC124276.2  GPR3  PNLIPRP3  NAALADL1  KRTAP2-3  LINC01537  NLRP3 | LINC01204  SERPINB2  PAQR9  MTATP8P1  KRTAP2-3  IL1RL1  LINC02273  MESP2  KPNA7  VASH2  PNLIPRP3  LINC02742  AC020928.1  AC011511.4  LINC01605  AADACP1  SPANXC  AC124276.2  MUC7  SPRR1B  INHBA  MYH16  MARCHF4  AC073130.3  MANCR  EVI2A  AC037198.1  TMPRSS11E  ADPGK-AS1  AC012435.2 | AC027796.3  ST13P19  AC010547.1  STIMATE-MUSTN1  SPANXC  AL157400.5  AC007192.1  GTF2IP7  FGB  CD36  GBGT1  AP000692.2  ANXA10  CDH16  AL049839.2  MGAM  NGF  F5  AC002480.1  AC069360.1  KMO  AC103691.1  LUARIS  GPNMB  KPNA7  DCAF4L1  ABCA1  AC092171.3  AC002350.2  FAM227A | ZSWIM8-AS1  AMTN  F2RL2  AL353997.3  CRYBB3  UPK1B  LRP4-AS1  CEP83-DT  AC017060.1  MIR2117HG  AURKC  MIAT  RN7SL4P  AC090970.2  KCNK3  AC010148.1  AC037198.1  APLN  P2RX5-TAX1BP3  ARPIN-AP3S2  RN7SL2  ZNF551  LRRN2  AL161756.1  AC007040.2  THBS1  CHI3L2  AC005050.1  SKIDA1  AC020915.1 | AMTN  SPX  ST13P19  LINC00862  OLAH  NEB  SYCE2  CNRIP1  FAM78A  F5  PLAC4  TRIML2  RIMS2  AC037198.1  NR2F1-AS1  CLDN16  NOG  AC079414.3  SULT1E1  AC005674.2  UCN2  ALPK2  PYGM  ARC  FAT4  CCN2  GFPT2  FSIP1  LINC00520  IGF2BP3 | AL139300.1  AC002094.1  STEAP2  TMEM164  GCNT1  CIPC  SERPINA3  APOLD1  ZNF707  SLC16A7  MAFG  PLEKHA2  BLOC1S5-TXNDC5  AC068234.1  AC139530.2 | ATP6V1G2-DDX39B  ATP5MF-PTCD1  HBB  ANXA8L1  ZNF546  MPZL1  GABPB1-AS1  RBMS2  AL136295.4  FRS2  NFE2L1  SVIL-AS1  SZRD1  CAB39  BLOC1S5-TXNDC5  DNAAF4-CCPG1  INO80B-WBP1  BIVM-ERCC5  TRIM39-RPP21 | AC010323.1  AC008764.1  BIVM-ERCC5  AC087289.3  JMJD7  CFHR3  CD274  AC016876.3  SYS1-DBNDD2  SNX15  KIAA0408  ISY1-RAB43  ST13P19  AL158066.1  AC068234.1  TNFSF12-TNFSF13 |

HBEs*^NC^* treated with vehicle and HBEs*^SPP1 KD^* treated with vehicle [HBEs^KD^ (Veh) vs HBEs *^NC^* (Veh) ];

HBEs*^NC^* treated with CSE and HBEs*^SPP1 KD^* treated with CSE [HBEs ^KD^(CSE)vs HBEs *^NC^*(CSE) ];

HBEs*^NC^* co-culture with TCs with vehicle and HBEs*^SPP1 KD^* co-culture with TCs with vehicle [HBEs ^KD^ /TC(Veh) vs HBEs ^NC^/TC(Veh)];

HBEs*^NC^* pretreated with CSE co-culture with TCs and HBEs *^SPP1 KD^* pretreated with CSE co-culture with TCs [HBEs ^KD^ (CSE)/TCs (Veh) vs HBEs^NC^ (CSE)/TCs (Veh)];

HBEs*^NC^* co-culture with TCs with CSE and HBEs *^SPP1 KD^* co-culture with TCs with CSE [HBEs ^KD^ (CSE)/TCs(CSE) vs HBEs ^NC^(CSE)/TCs(CSE)];

TCs co-culture with HBEs*^NC^* with vehicle and TCs co-culture with HBEs*^SPP1 KD^* with vehicle [TCs(Veh)/HBEs ^KD^ (Veh) vs TCs(Veh)/HBEs *^KD^* (Veh)];

TCs co-culture with HBEs*^NC^* pretreated with CSE and TCs co-culture with HBEs *^SPP1 KD^* pretreated with CSE [TCs(Veh)/HBEs ^KD^ (CSE) vs TCs(Veh)/HBEs^NC^(CSE)]

TCs co-culture with HBEs*^NC^* with CSE and TCs co-culture with HBEs *^SPP1 KD^* with CSE [TCs(CSE)/HBEs ^KD^ (CSE) vs TCs(CSE)/HBEs*^NC^*(CSE)].

Table 6. Down-regulation differentially expressed genes (DEGs) resulted from the RNA-seq data

| HBEs*^SPP1 OE^*(Veh)  vs HBEs*^NC^*(Veh) | HBEs^OE^ (CSE)  vs HBEs^NC^(CSE) | HBEs^OE^/TCs(Veh)  vs HBEs^NC^/TCs(Veh) | HBEs^OE^ (CSE)/TCs (Veh)  vs HBEs^NC^ (CSE)/TCs (Veh) | HBEs^OE^(CSE)/TCs(CSE)  vs HBEs^NC^(CSE)/TCs(CSE) | TCs(Veh)/HBEs^OE^(Veh)  vs TCs(Veh)/HBEs^NC^(Veh) | TCs(Veh)/HBEs^OE^(CSE)  vs TCs(Veh)/HBEs^NC^(CSE) | TCs(CSE)/HBEs^OE^(CSE)  vs TCs(CSE)/HBEs^NC^(CSE) |
| --- | --- | --- | --- | --- | --- | --- | --- |
| TMEM97  MTND4P12  SLC7A2  RAB43  PI15  RP11-511P7.5  AC087190.3  AC092143.1  PCDHGB4  ST13P19  BIVM-ERCC5 | RAPGEF5  STK4  CNOT9  ORAI3  SC5D  KRT17P2  IGKC  AL139353.1  RPL19P16  ISY1-RAB43  AC139530.2  BIVM-ERCC5  UGT1A8 | CLDN12  MAFG  AL662899.2  AP002990.1  TM4SF19-TCTEX1D2  ST13P19  TEX14 | NOTCH2NLA  AC010547.4  ST13P19  AC138811.2  KRT6C | SHISA5  ARMCX3  TTPAL  SNX15  PMF1-BGLAP  AP002990.1 | ZKSCAN7  ARL2-SNX15  YPEL2  AL139287.1  H3-2  AP002990.1  BIVM-ERCC5 | FAM102A  SPIN1  RDH5  SACS  AL139300.1  RP11-458D21.5  AC079594.2  AC078927.1  AC109635.3 | FYTTD1  TREX2  AC011462.1  AL158066.1 |

HBEs*^NC^* treated with vehicle and HBEs*^SPP1 OE^* treated with vehicle [HBEs^OE^ (Veh) vs HBEs *^NC^* (Veh) ];

HBEs*^NC^* treated with CSE and HBEs*^SPP1 OE^* treated with CSE [HBEs^OE^ (CSE)vs HBEs *^NC^*(CSE) ];

HBEs*^NC^* co-culture with TCs with vehicle and HBEs*^SPP1 OE^* co-culture with TCs with vehicle [HBEs ^OE^/TC(Veh) vs HBEs ^NC^/TC(Veh)];

HBEs*^NC^* pretreated with CSE co-culture with TCs and HBEs *^SPP1 OE^* pretreated with CSE co-culture with TCs [HBEs^OE^ (CSE)/TCs (Veh) vs HBEs^NC^ (CSE)/TCs (Veh)];

HBEs*^NC^* co-culture with TCs with CSE and HBEs *^SPP1 OE^* co-culture with TCs with CSE [HBEs ^OE^(CSE)/TCs(CSE) vs HBEs ^NC^(CSE)/TCs(CSE)];

TCs co-culture with HBEs*^NC^* with vehicle and TCs co-culture with HBEs*^SPP1 OE^* with vehicle [TCs(Veh)/HBEs^OE^(Veh) vs TCs(Veh)/HBEs^NC^(Veh)];

TCs co-culture with HBEs*^NC^* pretreated with CSE and TCs co-culture with HBEs *^SPP1 OE^* pretreated with CSE [TCs(Veh)/HBEs^OE^(CSE) vs TCs(Veh)/HBEs^NC^(CSE)]

TCs co-culture with HBEs*^NC^* with CSE and TCs co-culture with HBEs *^SPP1 OE^* with CSE [TCs(CSE)/HBEs^OE^(CSE) vs TCs(CSE)/HBEs^NC^(CSE)].

Table 7. Down-regulation differentially expressed genes (DEGs) resulted from the RNA-seq data

| HBEs^KD^(Veh)  vs HBEs^NC^(Veh) | HBEs^KD^(CSE)  vs HBEs^NC^(CSE) | HBEs^KD^/TC(Veh)  vs HBEs^NC^/TC(Veh) | HBEs^KD^ (CSE)/TC (Veh)  vs HBEs^NC^ (CSE)/TC (Veh) | HBEs^KD^(CSE)/TCs(CSE)  vs HBEs^NC^(CSE)/TCs(CSE) | TCs(Veh)/HBEs^KD^(Veh)  vs TCs(Veh)/HBEs^NC^(Veh) | TCs(Veh)/HBEs^KD^(CSE)  vs TCs(Veh)/HBEs^NC^(CSE) | TCs(CSE)/HBEs^KD^(CSE)  vs TCs(CSE)/HBEs^NC^(CSE) |
| --- | --- | --- | --- | --- | --- | --- | --- |
| MSLN  DHRSX  BCKDHB  PIGM  LINC01679  TRIM55  XG  IKBKE  TMSB15B  PCCA  KLHDC9  AC093627.4  TP53I3  AC106886.2  USP28  FYTTD1  SLC22A23  AC118344.1  GCNT4  LY6D  ZMIZ2  RP11-104N10.2  AMIGO1  STAT6  UCP2  PPP1R3E  PPM1J  PCED1A  CCDC57  ACOX1 | STARD4  HOXA-AS2  ZNF189  ZNF286A  EIF4HP2  ZNF596  USP28  RCOR3  MYO1G  SCD  TNFSF10  AC245140.2  CSPG4  MMP28  HERPUD2  HAGLR  ARHGAP30  FAM50B  A1BG-AS1  PCF11-AS1  EDARADD  CXXC5  PRPSAP2  RGL3  AFTPH  LHFPL2  ISYNA1  CLIC4P1  SH3TC1  NIPSNAP3B | CORO1A  MATN2  GLIPR2  TENT5B  TMEM92  DUSP1  RALGPS1  ENO1P4  TRPV3  EPB41L4A  IGFBP2  KRT16P6  MATN3  PPIL3  LMO2  SLC35E2A  BCL2A1  ANP32AP1  TUBAL3  ENTPD2  TLE2  CGNL1  CCNG2  ZCCHC24  FRY  TP53I11  PSCA  FAM25A  REEP1  ENTPD3 | IL1R2  PRSS22  SLC1A1  FGFBP1  NGEF  TMEM86A  RAB26  YWHAB  STXBP5-AS1  BAIAP3  CALCOCO1  S100A8  SNAI3  BICC1  CST6  MAIP1  IFFO1  RAET1G  GAS6  F3  LEPR  LGALS1  SLC2A3  TMEM92  TJP3  MX1  TMEM121  ORAI3  TTC9  AC018521.5 | FGF11  PLAT  PIGZ  CTSO  SCN5A  LY6G6C  ARHGAP33  ENPP5  COL4A5  ALDH3B2  RAPGEF3  ZNF513  SELENON  C6orf132  ANKRD20A5P  CASTOR3  P3H2  ALDH3B1  ESAM  DLX4  ZNF467  NLRP10  PLCG2  AC105020.1  FA2H  TMEM45B  GPR153  GDPGP1  LIF  ADAMTSL4 | CDC42SE2  GABPB1-AS1  KAZN  RASSF8  REPS2  THAP9  RBMS2  MPZL1  AP002990.1  AL049839.2  AC078927.1  PCDHGC4  RP11-458D21.5  AC011462.1 | SDC2  PCDH9  CDKN2AIP  CORO1C  RAPGEFL1  AC016876.3  MRFAP1  NBL1  HAVCR2  MDFIC  MXD1  EEF1E1-BLOC1S5  AC005943.1 | SZRD1  MED26  AC013394.1  ADIRF-AS1  HES1  PCDH9  AC138811.2  AMIGO3  AL662899.2  AL136295.1  AC137834.1  AC008695.1 |

HBEs*^NC^* treated with vehicle and HBEs*^SPP1 KD^* treated with vehicle [HBEs^KD^ (Veh) vs HBEs *^NC^* (Veh) ];

HBEs*^NC^* treated with CSE and HBEs*^SPP1 KD^* treated with CSE [HBEs ^KD^(CSE)vs HBEs *^NC^*(CSE) ];

HBEs*^NC^* co-culture with TCs with vehicle and HBEs*^SPP1 KD^* co-culture with TCs with vehicle [HBEs ^KD^ /TC(Veh) vs HBEs ^NC^/TC(Veh)];

HBEs*^NC^* pretreated with CSE co-culture with TCs and HBEs *^SPP1 KD^* pretreated with CSE co-culture with TCs [HBEs ^KD^ (CSE)/TCs (Veh) vs HBEs^NC^ (CSE)/TCs (Veh)];

HBEs*^NC^* co-culture with TCs with CSE and HBEs *^SPP1 KD^* co-culture with TCs with CSE [HBEs ^KD^ (CSE)/TCs(CSE) vs HBEs ^NC^(CSE)/TCs(CSE)];

TCs co-culture with HBEs*^NC^* with vehicle and TCs co-culture with HBEs*^SPP1 KD^* with vehicle [TCs(Veh)/HBEs ^KD^ (Veh) vs TCs(Veh)/HBEs *^KD^* (Veh)];

TCs co-culture with HBEs*^NC^* pretreated with CSE and TCs co-culture with HBEs *^SPP1 KD^* pretreated with CSE [TCs(Veh)/HBEs ^KD^ (CSE) vs TCs(Veh)/HBEs^NC^(CSE)]

TCs co-culture with HBEs*^NC^* with CSE and TCs co-culture with HBEs *^SPP1 KD^* with CSE [TCs(CSE)/HBEs ^KD^ (CSE) vs TCs(CSE)/HBEs*^NC^*(CSE)].
